# Combining biophysical parameters with thermal and RGB indices using machine learning models for predicting yield in yellow rust affected wheat crop

**DOI:** 10.1038/s41598-023-45682-3

**Published:** 2023-11-01

**Authors:** RN Singh, P. Krishnan, Vaibhav K. Singh, Sonam Sah, B. Das

**Affiliations:** 1 Division of Agricultural Physics, ICAR-Indian Agricultural Research Institute, New Delhi, India; 2 ICAR-National Institute of Abiotic Stress Management, Pune, Maharashtra India; 3 Division of Plant Pathology, ICAR-Indian Agricultural Research Institute, New Delhi, India; 4 ICAR-Central Coastal Agricultural Research Institute, Old Goa, Goa India

**Keywords:** Plant stress responses, Image processing, Machine learning

## Abstract

Evaluating crop health and forecasting yields in the early stages are crucial for effective crop and market management during periods of biotic stress for both farmers and policymakers. Field experiments were conducted during 2017–18 and 2018–19 with objective to evaluate the effect of yellow rust on various biophysical parameters of 24 wheat cultivars, with varying levels of resistance to yellow rust and to develop machine learning (ML) models with improved accuracy for predicting yield by integrating thermal and RGB indices with crucial plant biophysical parameters. Results revealed that as the level of rust increased, so did the canopy temperature and there was a significant decrease in crop photosynthesis, transpiration, stomatal conductance, leaf area index, membrane stability index, relative leaf water content, and normalized difference vegetation index due to rust, and the reductions were directly correlated with levels of rust severity. The yield reduction in moderate resistant, low resistant and susceptible cultivars as compared to resistant cultivars, varied from 15.9–16.9%, 28.6–34.4% and 59–61.1%, respectively. The ML models were able to provide relatively accurate early yield estimates, with the accuracy increasing as the harvest approached. The yield prediction performance of the different ML models varied with the stage of the crop growth. Based on the validation output of different ML models, Cubist, PLS, and SpikeSlab models were found to be effective in predicting the wheat yield at an early stage (55-60 days after sowing) of crop growth. The KNN, Cubist, SLR, RF, SpikeSlab, XGB, GPR and PLS models were proved to be more useful in predicting the crop yield at the middle stage (70 days after sowing) of the crop, while RF, SpikeSlab, KNN, Cubist, ELNET, GPR, SLR, XGB and MARS models were found good to predict the crop yield at late stage (80 days after sowing). The study quantified the impact of different levels of rust severity on crop biophysical parameters and demonstrated the usefulness of remote sensing and biophysical parameters data integration using machine-learning models for early yield prediction under biotically stressed conditions.

## Introduction

Wheat, scientifically known as *Triticum aestivum L*., is a highly significant staple crop with immense global economic value. Wheat holds the distinction of being the largest cultivated crop worldwide, occupying approximately 225 million hectares of land with a total production of around 772.64 million metric tons and a productivity rate of 3.25 tons per hectare^1^. Wheat plays a crucial role in providing an affordable source of protein (20%) and calories (19%) through consumption to majority of the world population^2^. Given its status as a staple food and its ability to cater to the needs of a large population, particularly those in poverty, wheat carries special importance in ensuring global food security. With the projected increase in the world's population, the demand for wheat is expected to rise by 60% by 2050^3^. India, being the second largest wheat producer globally, contributes approximately 13.5% pf the global wheat production^1^. According to an estimate 10–16% of world wheat produce is lost due to pest and diseases^4^.Yellow rust, also called stripe rust of wheat is considered most economically important disease of wheat and threat to world food security^5^. The yellow rust infection most commonly occurs on the wheat leaves, which reduces the light interception and photosynthesis resulting lower yields. Understanding and quantifying the changes in plant biophysical parameters in reponse to different levels of plant disease severity is essential for the effective management of resources.

Wang et al.^6^ studied the alterations in leaf area, photosynthetic rate, stomatal conductance, transpiration rate of oat to understand the plant response towards leaf blight disease. Francesconi and Balestra^7^ studied the changes in photosynthesis and stomatal conductance to describe wheat response for fusarium head blight disease. In order to comprehend how plants react to stressors, the relative leaf water content (RWC) is a significant parameter. Several researchers have employed RWC to examine how plants respond to various plant diseases, such as vascular wilt of tomato^8^, fusarium canker in almond^9^, root rot of pea^10^, and sharp eyespot and fusarium head blight of wheat^11^. Membrane stability index (MSI) is a widely used physiological index to evaluate plant stress under biotic and abiotic stressors^11^. The leaf area index (LAI) is another fundamental variable that has a strong correlation with crop photosynthesis and the accumulation of dry matter in crops and hence it is an important parameter for tracking the growth status of crops and predicting their yield^12,13^. Several researchers have used LAI as a key indicator for monitoring the growth conditions of crop like maize^14^, wheat^15^, rice^16^ etc. The Normalized difference vegetation index (NDVI) is a highly effective indicator of photosynthetically active biomass and is proficient at precisely discerning shifts in crop conditions^17^. It is also one of the most frequently utilized vegetation index and finds broad application in identifying nutrient deficiencies, indirect photosynthesis estimation, and the detection of both biotic and abiotic stress conditions^18^. Additionally, the NDVI is the most commonly employed vegetative index for identifying diseased tissue^19^. Several researcher have used NDVI for monitoring and evaluating impacts of yellow rust on wheat crop^3,20,21^.

Though researchers have explored various biophysical parameters of wheat under diseased conditions, a notable gap in these assessments is that they are predominantly focused on distinguishing only between diseased and non-diseased conditions. To enhance wheat yield and economic returns, a crucial step is to comprehend how plant biophysical parameters are affected by varying degrees of disease severity. Applying a uniform approach to combat yellow rust frequently leads to excessive chemical use, diminishing farmers' profits and causing environmental harm^5^. To best of our knowledge none of studies till date quantified plant biophysical responses in wheat cultivars under different levels of rust severity and hence there is a notable scarcity of research examining the effects of different yellow rust severity levels on diverse wheat cultivars under field conditions.

Predicting crop yield is another crucial task in the current scenario for policymakers and farmers in order to ensure food security and sustainability. Nevertheless, this task poses significant challenges due to the complex interrelationships among soil, plant, and environmental factors that impact crop yield^22^. The major limitations of traditional techniques of yield prediction such as crop growth models and statistical methods lies in their inability to effectively account for the constantly changing biotic and abiotic factors that affect crop production. Furthermore, these conventional models necessitate a substantial amount of data relating to soil, climate, crop, and management practices, as well as significant user proficiency and expertise to fine-tune the model^23^. The progress made in machine learning (ML) has introduced a novel and enhanced approach to overcome the above limitations in yield prediction of agricultural crops^24^. Several researchers used machine learning as well as deep learning approaches to predict yield in crops like wheat^25,26^, pigeon pea^27^, maize^28^, oil palm^29^ etc. under varying environmental conditions. However, researchers focusing on machine learning for yield prediction primarily rely on remote sensing data, which offers limited accuracy in early yield prediction. Augmenting remote sensing data with actual plant biophysical information has the potential to enhance the accuracy of early yield prediction. An evident research gap exists in the integrated utilization of plant biophysical data and image-based information for yield prediction. No prior study has undertaken the combination of thermal and visible image-derived indices with biophysical parameters to forecast wheat yield under biotic stress conditions.

With this background, the current study is planned to (a) investigate and quantify the effect of yellow rust on the biophysical parameters of wheat cultivars under different levels of yellow rust severity (b) coupling plant biophysical parameters data with image-based visible and thermal indices using ML models for improving yield prediction under biotically stressed conditions and (c) comparing the ability of different ML models to predict wheat yield under biotically stressed conditions.

## Materials and methods

**Figure 1:**
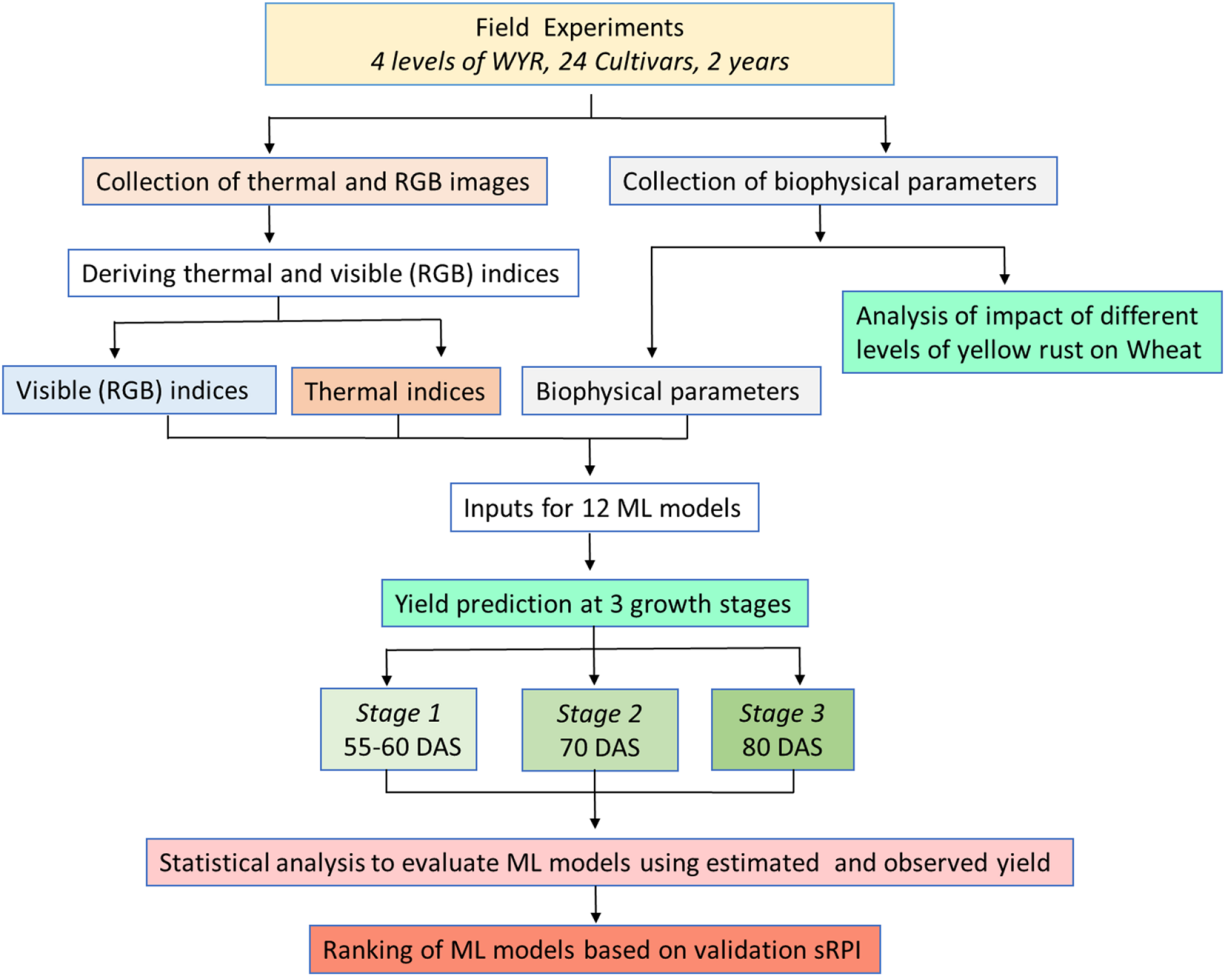
Flow chart describing overview of the methodology. *WYR* Wheat yellow rust, *ML models* Machine learning models, *DAS* Days after sowing, *sRPI* Standardized ranking performance index.

Thermal and visible images were collected for two years at different crop growth stages, along with biophysical parameters of the crop, including the LAI, RWC, MSI, photosynthesis, stomatal conductance, transpiration, and intercellular CO_2_ concentration. These measurements were taken alongside the NDVI. To compare the impact of yellow rust on different cultivar categories, we used a one-way ANOVA with a post hoc Tukey test at a significance level of 5%. The crop yields were predicted using ML models at 3 different growth stages by using image based indices and biophysical parameters as model inputs. The performances of ML models were evaluated using coefficient of determination (R^2^), d-index, root mean square error (RMSE), normalized root mean square error (n-RMSE) and mean bias error (MBE). Standardized ranking performance index (sRPI) derived from R^2^, d-index, RMSE and MBE of calibration and validation was utilized to finally rank models for their ability of yield prediction. A flow chart describing the methodology is presented in Fig. 1 and detailed methodology is elaborated in the subsequent sections.

### Study site, treatment details and recording yellow rust severity

Field experiments were conducted over a two-year period (2017–2018 and 2018–2019) at the Indian Agricultural Research Institute's research farm in New Delhi, which has a semi-arid climate with hot, dry summers and dry winters. The crop was grown from the middle of November to the first week of April. The soil at the experimental site is non-calcareous and slightly alkaline, classified as Indo-Gangetic alluvium (Typic *Haplustepts*). Twenty-four different wheat cultivars with varying levels of resistance and susceptibility were planted in plots measuring 2.5 × 2.5 m with a row spacing of 25 cm. The planting occurred on November 24th in the first year and November 25th in the second year. In order to create an artificial epiphytotic in field conditions, a mixture of six virulent and most predominant pathotypes of *P. striiformis tritici* were used as urediospore inoculum. Disease severity was recorded on a weekly basis after when susceptible checks reached 25–30% severity according to the modified Cobb's scale^30^, and the final disease severity (FDS) was taken for yellow rust on March 8th during the first year and March 4th during the second year. The cultivars were divided and classified into four different categories viz. High (Highly resistant, < 20% FDS), Moderate (moderately resistant, 20–40% FDS), Low (low resistant, 40–60% FDS) and Susceptible (> 60% FDS) based on FDS as described by Singh et al.^31^.

### Measurement of biophysical parameters

*LAI:* Plant Canopy Analyzer (LAI-2000, Li-COR Ltd, Nebraska, USA) was utilized to determine the LAI of the plants. To obtain the LAI of the plot, three internal subsamples were taken for each plot for each cultivar and the results were averaged^32^. The observations were taken at 1700 h IST, when the diffused radiation was maximum, with a quarter view cap on the sensor to exclude the operator and the other part of the hemispherical view. A detailed protocol for LAI measurement is given in Liu et al.^33^. In the 2017–18, measurements were taken at 55, 70, 80, 95, and 110 days after sowing (DAS), while during the 2018–19, LAI readings were recorded at 60, 70, 80, 92, and 105 DAS.

*NDVI:* NDVI data was collected using a handheld NDVI sensor (Green-Seeker™, N-Tech Industries, Inc., USA) between 1300 and 1400 h IST to minimize sun angle effects^34^. The Green-Seeker sensor estimates the NDVI values using of reflectance data in the red and near infrared regions of the electromagnetic spectrum. The sensor was swept over the middle section of the wheat plots of each cultivar and three readings were taken. The average NDVI value was then calculated for each wheat cultivar. In the 2017–18, measurements were taken at 55, 70, 80, and 95 DAS, while during the 2018–19 the NDVI readings were recorded at 60, 70, 80 and 92 DAS.

*RWC:* The RWC was determined using the method described by Barrs and Weatherley^35^. The second leaflet from top of the plants were collected carefully to avoid any damage. To prevent loss of any moisture from the leaves, the samples were kept in icebox and the fresh weight (F) of the leaves were taken as quickly as possible. Subsequently, the leaves were saturated by adding enough water and kept for 6 h for attaining a constant weight by absorbing water and the weight of the saturated leaf or the turgid weight (S) was recorded. Then the leaves were dried in hot air oven at 65 °C until constant dry weight (D) was obtained. Subsequently, using the F, D and S, RWC was calculated with the following formula: $RWC=(F-D)/(S-D)$ × 100. To get the average RWC value of a wheat cultivar, three internal replications were taken and averaged. In 2017–18, RWC measurements were taken at 55, 70, 80, and 95 DAS, while during 2018–19 the RWC readings were recorded at 60, 70, 80 and 92 DAS.

*MSI:* The measurement of MSI was conducted by taking 0.2 g of cut leaf samples and placing them in test tubes containing 20 ml of double-distilled water. These test tubes were then incubated at 30 °C overnight, and the electrical conductivity (EC) reading of the water containing the leaf samples was recorded as C_1_. Subsequently, the test tubes were subjected to boiling in a water bath at 100 °C for 15 min, followed by cooling to room temperature. The EC was again recorded for the solution containing the leaf samples as C_2_^36^. The MSI was then calculated using the equation : MSI (%) = (1 − (C_1_/C_2_)) × 100. As in the previous cases, three internal replications were taken for each cultivar and the values were averaged to obtain the average MSI values. In the 2017–18, MSI measurements were taken at 55, 70, 80, and 95 DAS, while during the 2018–19 the MSI readings were recorded at 60, 70, 80 and 92 DAS.

*Leaf photosynthesis, stomatal conductance, transpiration and intercellular CO*_*2*_* concentration:* Leaf photosynthesis rate, stomatal conductance, transpiration rate and intercellular CO_2_ concentration were measured using Infrared Gas Analyzer (IRGA, model LI-6400XT, Li-COR Ltd, Nebraska, USA). Mature leaflets of wheat were analyzed using an Infrared Gas Analyzer (IRGA, LI-6400XT, Li-COR Ltd, USA) under a constant light of 1000 µmol m^-2^ s^-1^. The IRGA was set to operate in closed method to measure leaf photosynthesis rate, stomatal conductance, transpiration rate, and intercellular CO_2_ concentration^37^. Three observations were taken for each cultivar on the uppermost completely extended leaves during the optimum period of photosynthetic active radiation from 1000 to 1230 h IST. The observations were recorded at 55, 70, 80, and 95 DAS in 2017–18, while during 2018–19 the readings were recorded at 60, 70, 80 and 92 DAS.

*Yield:* When the crops reached maturity, a manual harvest of 1 square meter was conducted. The grains were separated from the rest of the plant through threshing and beating and the yield was calculated based on the dry weight.

### Image acquisition and derived parameters

The thermal and visible images were acquired simultaneously using the Testo (890-1) handheld camera. This camera is equipped with thermal detector of resolution of 640 × 480 pixels and a high-quality wide-angle lens that covers a field of view of 42° × 32°. With a thermal sensitivity of less than 40 mK at 86 °F, the camera can detect even subtle temperature variations. It has a minimum focusing distance of 0.1 m. The camera's geometric resolution is 1.13 mrad when the emissivity is set at 0.95, and it operates within a spectral range of 8–14 µm. The temperature estimation capability of the camera ranges from − 20 to 100 °C, with an accuracy of ± 2%. The thermal and visible images were taken each year at four different dates between stem elongation to flowering period of the crop viz. 55, 70, 80 and 95 DAS in 2017–18 and 60, 70, 80 and 92 DAS in 2018–19. Images were captured from a nadir view angle at peak daytime temperatures and low wind conditions between 1330 and 1430 h IST^38^. Thermal and visible images were taken at three different locations in each plot from 1 m above the crop canopy, during various stages of crop growth. In the 2017–18, the images were taken at 55, 70, 80, and 95 DAS, while during the 2018–19 the images were captured at 60, 70, 80 and 92 DAS.

*Canopy temperature:* The average temperature of the canopy at various crop growth stages was derived using the thermal images. Testo IR soft analysis software was used to calculate the average temperature of the canopy by averaging the temperature values of each pixel in the image^39^.

*Visible and thermal indices:* Visible indices were calculated by extracting RGB tristimulus values using R software version 4.1.2. The RGB values were normalized in order to minimize the impact of illumination and variations in color^40^. Thermal indices were computed by utilizing temperature values extracted from the pixel data of thermal images using Testo IR software, in conjunction with the concurrent measurement of wet and dry leaf temperatures taken while capturing the thermal image^41,42^. We generated 45 visible and 3 thermal indices for each of the three stages for yield prediction. These indices along with their respective formulas are provided in Supplementary Table 1.

### ML models for predicting yield

The biophysical parameters and the image based indices of first three dates of observations of each year were used to develop 12 ML models, which include Elastic Net (ELNET), Support Vector Machine (SVM), Gaussian Process Regression (GPR), Generalized Linear Model (GLM), Spike and Slab Regression (SpikeSlab), Multivariate Adaptive Regression Spline (MARS), Partial Least Square Regression (PLS), Random Forest (RF), K-Nearest Neighbours (KNN), Stepwise Linear Regression (SLR), eXtreme Gradient Boosting (XGB), and Cubist. Details on the ML models used in this study is also described in Singh et al.^42^. For yield prediction, we utilized dataset generated from collecting data of 24 cultivars at three growth stages resulting 48 data points for calibration and validation at each stage. The predictor variables for yield included RGB and thermal image indices, LAI, RWC, NDVI, MSI, photosynthesis rate, stomatal conductance, transpiration rate, intercellular CO_2_ concentration and image derived canopy temperature. Out of the total data at available at each stage, 70% of the data points were used in calibration and the remaining 30% were used for validation. The parameters of the ML models were fine-tuned and adjusted through tenfold cross-validation with five repetitions, employing the 'caret' package^43^ in R software version 4.1.2^44^. To evaluate the predictive capability of the models several statistical parameters including the R^2^, d-index, MBE, RMSE and n-RMSE were calculated. n-RMSE (%) values within the range of 0–10% are considered “excellent”, those between 10–20% are classified as “good”, values from 20–30% fall into the “fair” category, and any values exceeding 30% are deemed “poor”^45,46^. To rank the models by combining the results of calibration and validation of multiple statistical criteria, standardized Ranking Performance Index (sRPI) was calculated^47^. The sRPI ranges between 0 and 1, where the worst-performing model gets 0, while the best model gets value of 1. The yield predictions were made at three different stages before crop harvest viz, Stage 1: 55–60 DAS, Stage 2: 70 DAS, and Stage 3: 80 DAS.

### Ethical approval

The planting material used in this study was procured from the Division of Plant Pathology, ICAR-Indian Agricultural Research Institute, New Delhi, India. We confirm that the current study complies with relevant institutional, national, and international guidelines and legislation for experimental research and field studies on plants (either cultivated or wild), including the collection of plant materials.

## Results

### FDS in wheat cultivars

The FDS in twelve wheat cultivars ( PDW 314, WH 1124, DBW 90, HD 3086, PDW 291, HS 507, VL 907, VL 829, HI 1563, HD 3043, PBW 644 and, Raj 4083) remained below 20% which are categorized as highly resistant cultivars. Six wheat cultivars (HPW 349, HD 2932, WH 1105, DBW 88, HS 375 and HD 3059) have FDS between 21 and 40% and these are categorized as moderate resistant cultivars. Similarly, three cultivars (HPW 251, HD 2967 and PBW 590) have FDS between 41 and 60%, and are categorized as low resistance cultivars. Finally, in three cultivars (HS 240, PBW 343 and A-9-30-1) the FDS was more than 80% during both years and they are categorized as susceptible cultivars (Supplementary Table 2).

### Variations in wheat biophysical parameters under different levels of yellow rust severity

As rust severity levels increase, there is a consistent decrease in all biophysical parameters, such as LAI, NDVI, MSI, RWC, leaf photosynthesis rate, stomatal conductance, transpiration rate, and yield, with the exception of canopy temperature. At all growth stages, the average values of these parameters were highest for cultivars categorized as high resistant, followed by moderate resistant and low resistant, while the least values were observed in susceptible cultivars. The mean values of biophysical parameters of each group of cultivars is presented in Table 1. The graphical representations were also provided in Supplementary Figs. 1, 2, 3, 4, 5, 6, 7, 8 and 9. The individual parameters were discussed briefly in the following sections.

**Table 1:**
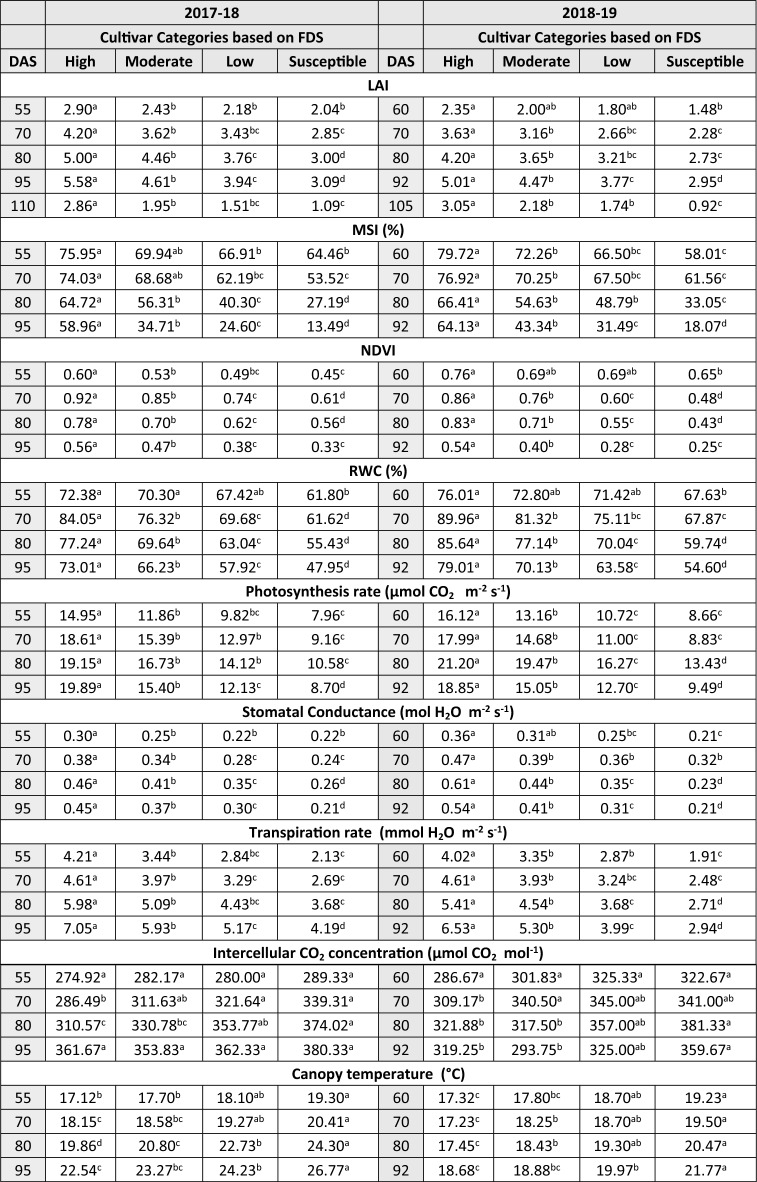
Effect of different levels of wheat yellow rust severity on different categories of wheat cultivars.

*Letters indicates statistically significant differences between groups, with shared letters implying similarity and different letters indicating dissimilarity in means.

*LAI:* The LAI of the wheat cultivars decreases as the level of yellow rust severity increases and differs significantly at all growth stages in both years. The category wise highest average values of LAI achieved in high, moderate, low and susceptible cultivars were 5.57, 4.64, 3.93 and 3.09, respectively, at 95 DAS in 2017–18, whereas in 2018–19 the maximum values of LAI for high, moderate, low and susceptible category cultivars were 5.01, 4.47, 3.76 and 2.95, respectively at 92 DAS in 2018–19. The trend of LAI variation in the order of High > Moderate > Low > Susceptible remained consistent in both years across all growth stages. Considering the average of reductions as compared to resistant category in both years, the LAI at 55–60 DAS decreased by 15.52%, 23.99%, and 33.30% for moderate, low, and susceptible category cultivars, respectively. Similarly, at 70 DAS, these reductions averaged 13.51%, 22.57%, and 34.73%, and by 80 DAS, they were 11.93%, 24.23%, and 37.49%, respectively. Moving forward to 92–95 DAS, the average LAI reductions were 14.02%, 27.14%, and 42.84%, while at 105–110 DAS, the reductions were around 30.35%, 45.12%, and 65.82% for moderate, low, and susceptible category cultivars, respectively (Table 1).

*NDVI:* The NDVI of the wheat cultivars also decreases as the level of yellow rust severity increases and differs significantly at all growth stages in both years. The maximum values of NDVI achieved in high, moderate, low and susceptible category cultivars were 0.93,0.85, 0.74 and 0.61, respectively, at 70 DAS in 2017–18, whereas in 2018–19 the maximum values of NDVI for high, moderate, low and susceptible category cultivars were 0.85, 0.76, 0.69 and 0.65, respectively at 70 DAS. The trend of NDVI variation in the order of High > Moderate > Low > Susceptible in both years was same at all growth stages. Considering the average of reductions as compared to resistant category in both years, the NDVI at 55–60 DAS decreased by 10.47%, 13.22%, and 19.63% for moderate, low, and susceptible category cultivars, respectively. Likewise, at 70 DAS, these reductions averaged 9.84%, 24.66%, and 39.82%, and by 80 DAS, they amounted to 12.46%, 26.92%, and 38.13%. Progressing to 92–95 DAS, the average NDVI reductions were 21.80%, 40.79%, and 46.86% for moderate, low, and susceptible category cultivars, respectively (Table 1).

*MSI:* The MSI of the wheat cultivars also decreases as the level of yellow rust severity increases and differs significantly at all growth stages in both years. The MSI values were higher during  disease initiation and decreased gradually with the progress of the yellow rust and crop maturity. The maximum values of MSI achieved in high, moderate, low and susceptible category cultivars were 75.94, 69.94, 66.91, and 64.45%, respectively, at 55 DAS in 2017–18. In the second year (2018–19), the maximum values of MSI for high and moderate category cultivars were 79.72 and 72.76%, respectively at 60 DAS, while for low and susceptible category cultivars the maximum values of MSI were 67.50 and 61.56%, respectively at 70 DAS. The trend of MSI variation in both years was same at all growth stages viz. High > Moderate > Low > Susceptible. In both years, when comparing with the resistant category cultivars, the MSI also displayed significant reductions across various growth stages.

Considering the average of reductions in both years, at 55–60 DAS, the MSI declined by 8.63%, 14.24%, and 21.18% for moderate, low, and susceptible category cultivars, respectively. Similarly, at 70 DAS, these reductions averaged 7.95%, 14.12%, and 23.84%, and by 80 DAS, the reductions were 15.37%, 32.14%, and 54.11%, respectively. Progressing to 92–95 DAS, the average MSI reductions were 36.78%, 54.58%, and 74.47% for moderate, low, and susceptible category cultivars, respectively (Table 1).

*Photosynthesis rate:* The photosynthesis rate in the wheat cultivars was found to be decreasing with increasing level of yellow rust severity and differs significantly at all growth stages in both years. During 2017–18, the maximum values of photosynthesis rate achieved in high resistant cultivars was 19.89 µmol CO_2_ m^−2^ s^−1^ at 95 DAS, while for moderate, low and susceptible category cultivars the maximum values of photosynthesis rate were 16.72, 14.11 and 10.58 µmol CO_2_ m^−2^ s^−1^, respectively at 80 DAS. In 2018–19, the maximum values of photosynthesis rate for high, moderate, low and susceptible category cultivars were 21.20, 19.46, 16.26 and 13.43 µmol CO_2_ m^−2^ s^−1^, respectively at 80 DAS. The trend of photosynthesis rate in both years was same at all growth stages viz., High > Moderate > Low > Susceptible. Considering the average of reductions as compared to resistant category in both years the photosynthesis rate at 55–60 DAS decreased by 19.49%, 33.90%, and 46.54% for moderate, low, and susceptible category cultivars, respectively. Likewise, at 70 DAS, these reductions averaged 17.87%, 34.60%, and 50.84%, and by 80 DAS, they amounted to 10.42%, 24.78%, and 40.68%. Progressing to 92–95 DAS, the average photosynthesis rate reductions were 21.37%, 35.82%, and 52.97% for moderate, low, and susceptible category cultivars, respectively (Table 1).

*Stomatal conductance:* The stomatal conductance in the wheat cultivars was found to be decreasing with increasing level of yellow rust severity. At initial stage of observations (55–70 DAS), the adjacent categories were somewhat similar statistically, however after 80 DAS all the categories were significantly different at 5% level of significance in both years. During 2017–18, the maximum values of stomatal conductance achieved in high, moderate, low and susceptible category cultivars were 0.456, 0.408, 0.346 and 0.262 mol H_2_O m^−2^ s^−1^, respectively at 80 DAS. Similarly, in 2018–19, the maximum values of photosynthesis rate for high and moderate category cultivars were 0.609 and 0.435 mol H_2_O m^−2^ s^−1^, respectively at 80 DAS, while for low and susceptible category the maximum values were 0.363 and 0.316 mol H_2_O m^−2^ s^−1^, respectively at 70 DAS. The trend of stomatal conductance in both years was same at all growth stages viz., High > Moderate > Low > Susceptible. The maximum difference in stomatal conductance of high resistance and susceptible cultivars was observed at 95 DAS and at 80 DAS in 2017–18 and 2018–19, respectively (Table 1). Considering the average of reductions as compared to resistant category in both years the stomatal conductance at 55–60 DAS decreased by 14.80%, 28.04%, and 34.60% for moderate, low, and susceptible category cultivars, respectively. Similarly, at 70 DAS, these reductions averaged 14.96%, 25.77%, and 34.87%, and by 80 DAS, they were 19.49%, 33.28%, and 52.07%. Moving forward to 105–110 DAS, reductions were around 21.67%, 38.95%, and 57.87% for moderate, low, and susceptible category cultivars, respectively.

*Transpiration rate:* The transpiration rate in the wheat cultivars was found to be decreasing with increasing level of yellow rust severity and differs significantly at all growth stages in both years. During 2017–18, the maximum values of transpiration rates in high, moderate, low and susceptible category cultivars were 7.045, 5.932, 5.167, and 4.193 mmol H_2_O m^−2^ s^−1^, respectively at 95 DAS. In 2018–19, the maximum values of transpiration rate for high, moderate, low and susceptible category cultivars were 6.527, 5.295, 3.990, and 2.943 mmol H_2_O m^−2^ s^−1^, respectively at 92 DAS. The trend of stomatal conductance in both years was same at all growth stages viz., High > Moderate > Low > Susceptible. Considering the average of reductions as compared to resistant category in both years, the transpiration rate at 55–60 DAS declined by 17.47%, 30.59%, and 50.97% for moderate, low, and susceptible category cultivars, respectively. Similarly, at 70 DAS, these reductions averaged 14.37%, 29.13%, and 43.88%, and by 80 DAS, the reductions were 15.49%, 28.87%, and 44.15%, respectively. Progressing to 92–95 DAS, the average reductions in transpiration rate were 17.34%, 32.76%, and 47.69% for moderate, low, and susceptible category cultivars, respectively. (Table 1).

*Intercellular CO*_*2*_* concentration:* The intercellular CO_2_ concentration was found to be increasing as the level of rust severity increases. However, in most of the cases the differences are not statistically significant and the trend is also irregular. During 2017–18, the maximum values of intercellular CO_2_ concentration in high, moderate, low and susceptible category cultivars were 361, 353, 362 and 380 µmol CO_2_ mol^−1^, respectively at 95 DAS. In 2018–19, the maximum values of intercellular CO_2_ concentration for high, low and susceptible category cultivars were 321, 357 and 381 µmol CO_2_ mol^−1^, respectively at 80 DAS, while for moderate resistant cultivar group the intercellular CO_2_ concentration had maximum value of 340 µmol CO_2_ mol^−1^ at 70 DAS. Considering the average of reductions as compared to resistant category in both years the intercellular CO_2_ concentrations at 55–60 DAS increased by 3.96%, 7.67%, and 8.90% for moderate, low, and susceptible category cultivars, respectively. Likewise, at 70 DAS, these increments averaged 9.46%, 11.93%, and 14.37%, and by 80 DAS, they amounted to 2.57%, 12.41%, and 19.45% for moderate, low, and susceptible category cultivars, respectively. (Table 1).

*RWC:* The RWC of the wheat cultivars decreases as the level of yellow rust severity increases and differs significantly at all growth stages in both years. In 2017–18, the maximum values of RWC observed in high, moderate and low category cultivars were 84.50, 76.32, and 69.68%, respectively, at 70DAS, while in the susceptible category cultivars the maximum RWC was 61.80%, which was observed on 55 DAS. In the second year (2018–19) the maximum values of RWC for high, moderate, low and susceptible category cultivars were 89.55, 81.31, 75.11 and 67.86%, respectively at 70 DAS. The trend of RWC variation in both years was same at all growth stages viz. High > Moderate > Low > Susceptible. Considering the average of reductions as compared to resistant category in both years the RWC at 55–60 DAS decreased by 3.56%, 6.45%, and 12.82% for moderate, low, and susceptible category cultivars, respectively. Likewise, at 70 DAS, these reductions averaged 9.40%, 16.80%, and 25.62%, and by 80 DAS, they amounted to 9.88%, 18.3%, and 29.23%, respectively. Progressing to 92–95 DAS, the average RWC reductions were 10.27%, 20.1%, and 32.61% for moderate, low, and susceptible category cultivars, respectively (Table 1).

### Variations in average canopy temperature of wheat under different levels of yellow rust severity

The average canopy temperature increases with yellow rust severity levels and differs significantly among cultivars of all categories in both years. In both years, the trend of average canopy temperature in high, moderate, low and susceptible category cultivars was same at all growth stages viz., High < Moderate < Low < Susceptible. The maximum difference in average canopy temperature of high resistance and susceptible cultivars was 4.4 °C at 80 DAS and 3.1 °C at 92 DAS in 2017–18 and 2018–19, respectively. Considering the average of reductions as compared to resistant category in both years the average canopy temperature at 55–60 DAS, was higher by 3.1%, 6.87%, and 11.91% for moderate, low, and susceptible category cultivars, respectively. Similarly, at 70 DAS, the canopy temperature were higher by 4.14%, 7.33%, and 12.8%, while at 80 DAS they registered an increase of 5.19%, 12.54%, and 19.83%, respectively. Progressing to 92–95 DAS, the average canopy temperatures were higher by 2.17%, 7.21%, and 17.65% for moderate, low, and susceptible category cultivars, respectively (Table 1).

### Variations in wheat yield under different levels of yellow rust severity

**Figure 2:**
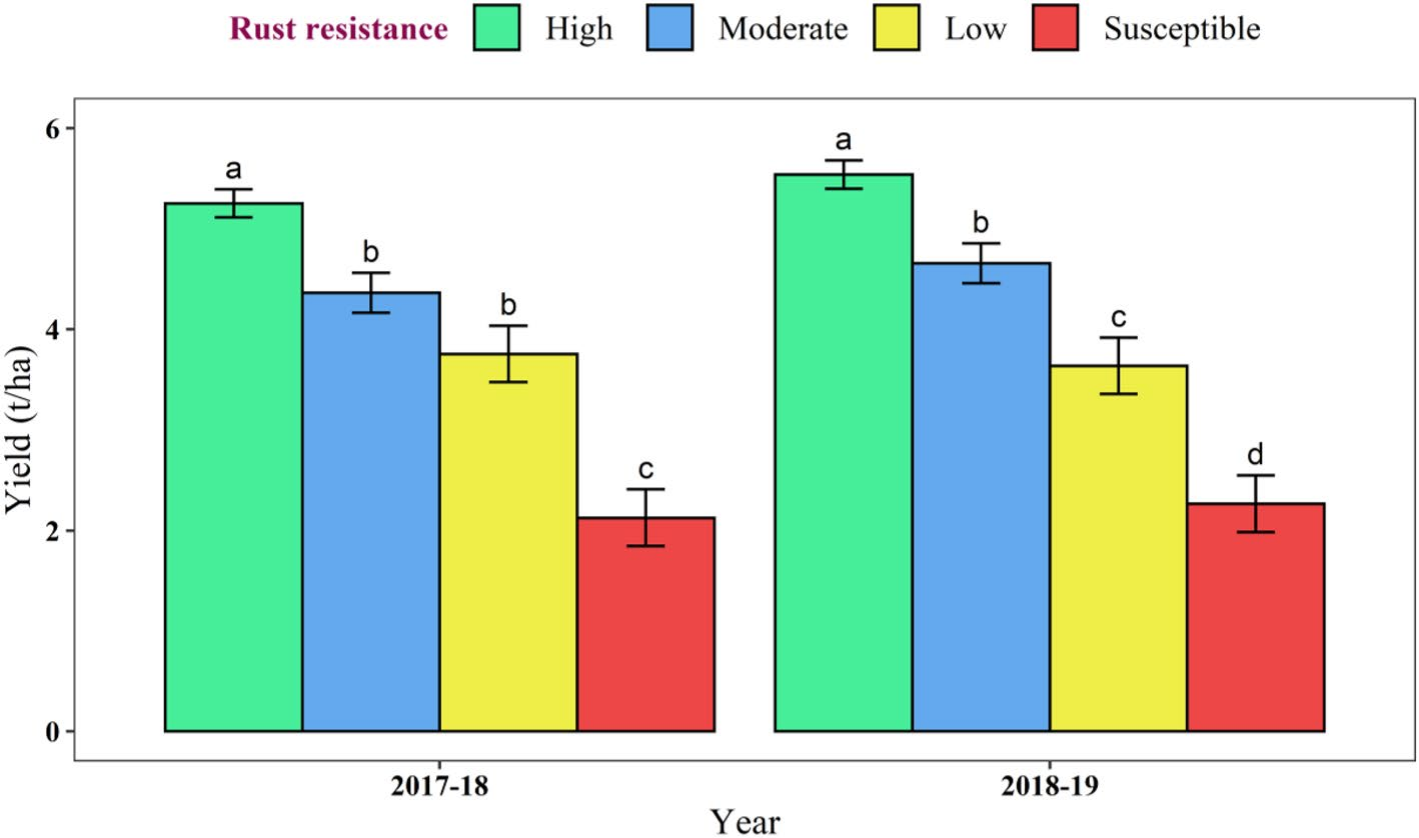
Yield variations in wheat cultivars under different levels of wheat yellow rust. Letters indicates statistically significant differences between groups, with shared letters implying similarity and different letters indicating dissimilarity in means.

The average group wise yield data indicated that the wheat yellow rust caused significant reduction in crop yield. In 2017–18, the average yields of high, moderate and low category cultivars were 5.25, 4.36, 3.75 and 2.04 t/ha, respectively. The yield of moderate resistant and low resistant category cultivars was statistically at par. However, significant differences are observed in rest of the cases. The yield reduction in the moderate, low and susceptible category cultivars were 16.96, 28.57, and 61.14%, respectively as compared to the high resistant cultivars. In 2018–19, the average yields of high, moderate and low category cultivars were 5.54, 4.66, 3.63 and 2.27 t/ha, respectively. The yield of all four-category cultivars were significantly different. The yield reduction in the moderate, low and susceptible category cultivars were 15.94, 34.42, and 59.09%, respectively as compared to the high resistant cultivars (Fig. 2).

### Performances of ML models to predict yield under different levels of yellow rust severity

#### Yield prediction using stage 1 data (55–60 DAS)

**Figure 3:**
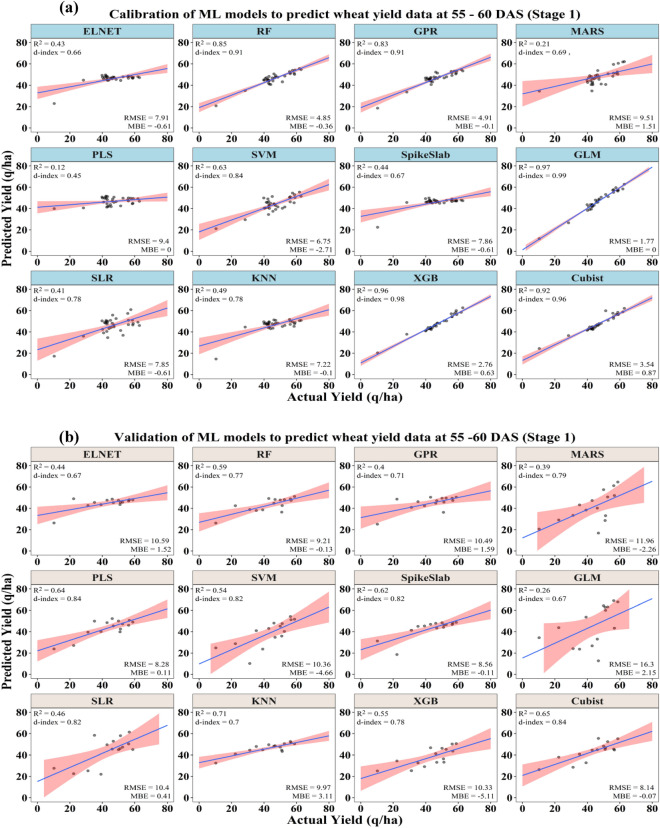
Performances of ML models to predict wheat yield under different yellow rust severity levels at 55–60 DAS after (**a**) calibration and (**b**) validation.

Using the data collected at 55 DAS in 2017–18 and at 60 DAS in 2018–19, some of the ML models were able to predict the wheat yield with acceptable accuracies (Table 2 and Fig. 3a,b). The calibration output indicated that among the different ML models, the GLM model was found to be more accurate in prediction of the crop yield with the highest coefficient of determination (R^2^) of 0.97, the maximum model agreement index (d-index) of 0.99 and the lowest RMSE of 1.77 q/ha. Based on the n-RMSE value (3.78%), the prediction accuracy of this model comes under the category of excellent prediction (0–10%). The PLS model was found to be the poorest among all in terms of crop yield prediction with the lowest coefficient of determination (R^2^) of 0.12, the least model agreement index (d-index) of 0.45, and a maximum RMSE of 9.40 q/ha. Based on the n-RMSE value (20.09%), the prediction accuracy of this model comes under the category of fair prediction (20–30%). Considering the prediction accuracy indicator (n-RMSE), the models GLM, XGB, and Cubist come under excellent prediction class (0–10%), ELNET, RF, GPR, SVM, SpikeSlab, SLR and KNN models come under good prediction class (10–20%) and MARS and PLS models come under fair prediction class (20–30%).

**Table 2:** Performance of different ML models for prediction of crop yield after calibration and validation using different biophysical parameters along with RGB and thermal image derived indices at 55–60 DAS.

	Calibration					Validation				
Models	R^2^	MBE	RMSE (q/ha)	n-RMSE (%)	d-index	R^2^	MBE	RMSE (q/ha)	n-RMSE (%)	d-index
ELNET	0.43	− 0.61	7.91	16.91	0.66	0.44	1.52	10.59	24.41	0.67
RF	0.85	− 0.36	4.85	10.37	0.91	0.59	− 0.13	9.21	21.25	0.77
GPR	0.83	− 0.10	4.91	10.49	0.91	0.40	1.59	10.49	24.20	0.71
MARS	0.21	1.51	9.51	20.32	0.69	0.39	− 2.26	11.96	27.58	0.79
PLS	0.12	0.00	9.40	20.09	0.45	0.64	0.11	8.28	19.09	0.84
SVM	0.63	− 2.71	6.75	14.43	0.84	0.54	− 4.66	10.36	23.90	0.82
SpikeSlab	0.44	− 0.61	7.86	16.80	0.67	0.62	− 0.11	8.56	19.73	0.82
GLM	0.97	0.00	1.77	3.78	0.99	0.26	2.15	16.30	37.58	0.67
SLR	0.41	− 0.61	7.85	16.78	0.78	0.46	0.41	10.40	23.97	0.82
KNN	0.49	− 0.10	7.22	15.44	0.78	0.71	3.11	9.97	22.98	0.70
XGB	0.96	0.63	2.76	5.89	0.98	0.55	− 5.11	10.33	23.82	0.78
Cubist	0.92	0.87	3.54	7.57	0.96	0.65	− 0.07	8.14	18.76	0.84

The validation output of different ML models in terms of the prediction of crop yields is presented in Table 2. Based on the model prediction error indicator (n-RMSE), the Cubist (18.76%), PLS (19.09%), and SpikeSlab (19.73%) models are categorized under the excellent prediction class (0–10%). The RMSE of these models ranged from 8.14 to 8.56 q/ha and the model agreement index (d-index) ranged from 0.82 to 0.84. Further, the GLM model was found to be the poorest among all with the highest percent of model prediction error (n-RMSE) of 37.58% and RMSE of 16.30 q/ha comes under the poor prediction category (n-RMSE > 30%). The rest of the models were categorized as fair prediction classes (n-RMSE between 20 and 30%).

#### Yield prediction using stage 2 data (70 DAS)

**Figure 4:**
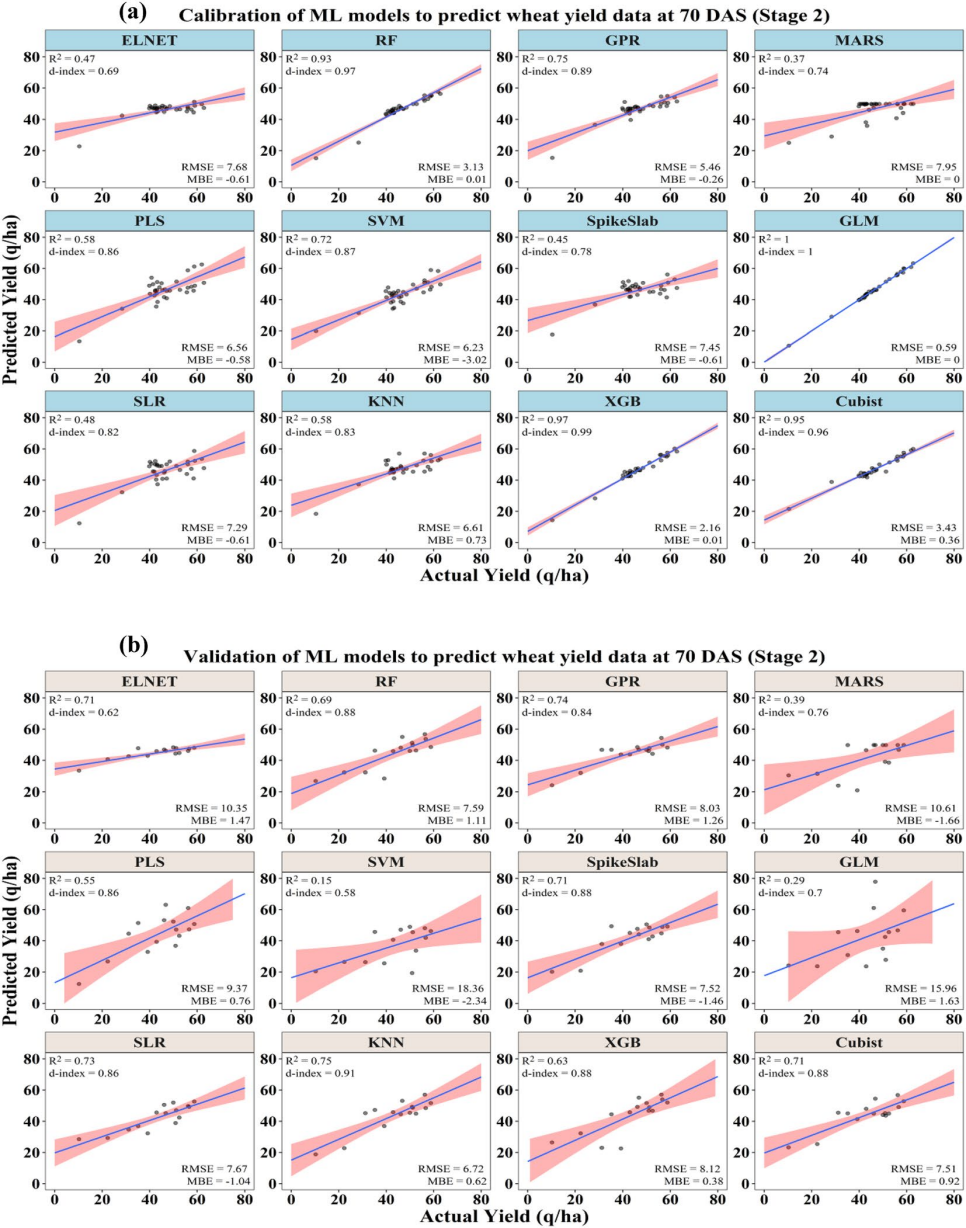
Performances of ML models to predict wheat yield under different yellow rust severity levels at 70 DAS after (**a**) calibration and (**b**) validation.

Using the data collected at 70 DAS in 2017–18 and 2018–19, the ML models were able to predict the wheat yield with higher accuracies as compared to the yield predictions using stage 1 (55–60 DAS) data. The model calibration output using stage 2 data was presented in Table 3 and depicted in Fig. 4a,b. The calibration output indicated that, among the different ML models, the GLM model was found to be more accurate in prediction of the crop yield with the highest coefficient of determination (R^2^) of 1.00, the maximum model agreement index (d-index) of 1.00 and the lowest RMSE of 0.59 q/ha. Based on the n-RMSE value (1.27%), the prediction accuracy of this model comes under the category of excellent prediction (0–10%). The MARS model was found to be the poorest among all in terms of crop yield prediction with the lowest coefficient of determination (R^2^) of 0.37, and a maximum RMSE of 7.95 q/ha. Considering the prediction accuracy indicator (n-RMSE), the models GLM, XGB, RF, and Cubist come under the excellent prediction class (0–10%) and the rest of the models come under the good prediction class (10–20%).

**Table 3:** Performance of different ML models in prediction of wheat yield after calibration and validation using different biophysical parameters along with RGB and thermal image derived indices at 70 DAS.

Models	Calibration					Validation				
	R^2^	MBE	RMSE (q/ha)	n-RMSE (%)	d-index	R^2^	MBE	RMSE (q/ha)	n-RMSE (%)	d-index
ELNET	0.47	− 0.61	7.68	16.42	0.69	0.71	1.47	10.35	23.87	0.62
RF	0.93	0.01	3.13	6.69	0.97	0.69	1.11	7.59	17.51	0.88
GPR	0.75	− 0.26	5.46	11.66	0.89	0.74	1.26	8.03	18.50	0.84
MARS	0.37	0.00	7.95	16.98	0.74	0.39	− 1.66	10.61	24.46	0.76
PLS	0.58	− 0.58	6.56	14.01	0.86	0.55	0.76	9.37	21.59	0.86
SVM	0.72	− 3.02	6.23	13.31	0.87	0.15	− 2.34	18.36	42.33	0.58
SpikeSlab	0.45	− 0.61	7.45	15.92	0.78	0.71	− 1.46	7.52	17.33	0.88
GLM	1.00	0.00	0.59	1.27	1.00	0.29	1.63	15.96	36.79	0.70
SLR	0.48	− 0.61	7.29	15.57	0.82	0.73	− 1.04	7.67	17.68	0.86
KNN	0.58	0.73	6.61	14.14	0.83	0.75	0.62	6.72	15.50	0.91
XGB	0.97	0.01	2.16	4.61	0.99	0.63	0.38	8.12	18.72	0.88
Cubist	0.95	0.36	3.43	7.33	0.96	0.71	0.92	7.51	17.31	0.88

The validation output of different ML models in terms of the prediction of crop yields using the stage 2 data set is presented in Table 3. Based on the model prediction error indicator (n-RMSE), the RF, GPR, SpikeSlab, SLR, KNN and XGB models are categorized under the excellent prediction class (0–10%). The RMSE of these models ranged from 7.52 to 8.12 q/ha and the model agreement index (d-index) ranged from 0.86 to 0.91. Further, the models SVM and GLM were found to be the poorest among all with the highest percent of model prediction error (n-RMSE) of 42.33% and 36.79% and RMSE of 18.36 q/ha and 15.96 q/ha, respectively. The rest of the models were categorized as fair prediction class (n-RMSE between 20 and 30%).

#### Yield prediction using stage 3 data (80 DAS)

**Figure 5:**
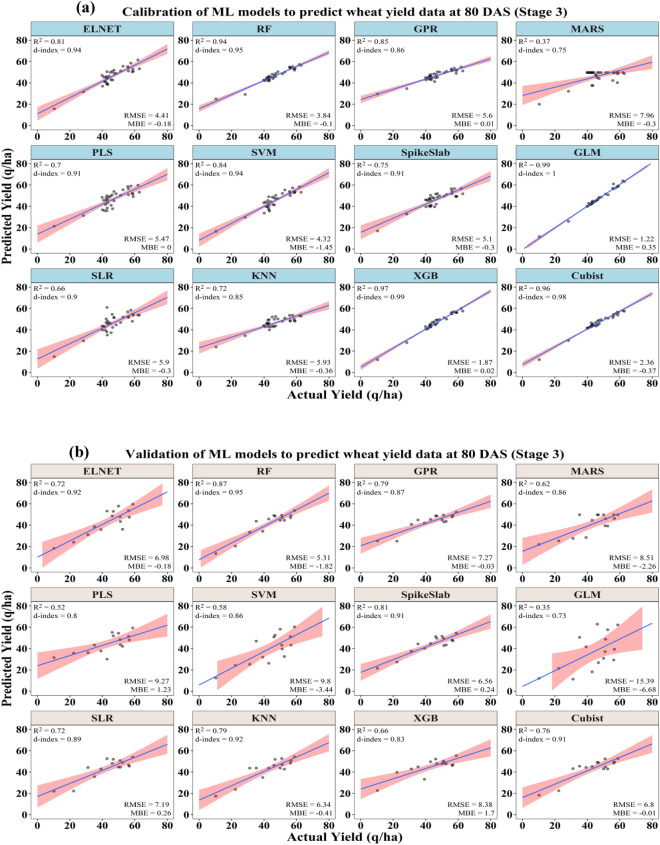
Performances of ML models to predict wheat yield under different yellow rust severity levels at 80 DAS after (**a**) calibration and (**b**) validation.

Using the data collected at 80 DAS in 2017–18 and 2018–19, the prediction accuracies of ML models were further improved as compared to the yield predictions made using data of 55–60 and 70 DAS. The model calibration output using stage 3 data was presented in Table 4 and depicted in Fig. 5a,b. The calibration output using stage 3 crop data revealed that, among the different ML models, the GLM model consistently proved to be more accurate in prediction of the crop yield with the highest coefficient of determination (R^2^) of 0.99, the maximum model agreement index (d-index) of 1.00 and the lowest RMSE of 1.22 q/ha. Based on the n-RMSE value (2.62%), the prediction accuracy of this model comes under the category of excellent prediction (0–10%). Considering the prediction accuracy indicator (n-RMSE), the models GLM, ELNET, RF, SVM,XGB, and Cubist can be categorized as the excellent prediction class (0–10%) and the rest of the models come under the good prediction class (10–20%).

**Table 4:** Performance of different ML models for prediction of crop yield after calibration and validation using different biophysical parameters along with RGB and thermal image derived indices at 80 DAS.

Models	Calibration					Validation				
	R^2^	MBE	RMSE (q/ha)	n-RMSE (%)	d-index	R^2^	MBE	RMSE (q/ha)	n-RMSE (%)	d– index
ELNET	0.81	− 0.18	4.41	9.43	0.94	0.72	− 0.18	6.98	16.09	0.92
RF	0.94	− 0.10	3.84	8.21	0.95	0.87	− 1.82	5.31	12.23	0.95
GPR	0.85	0.01	5.60	11.97	0.86	0.79	− 0.03	7.27	16.77	0.87
MARS	0.37	− 0.30	7.96	17.01	0.75	0.62	− 2.26	8.51	19.62	0.86
PLS	0.70	0.00	5.47	11.70	0.91	0.52	1.23	9.27	21.38	0.80
SVM	0.84	− 1.45	4.32	9.23	0.94	0.58	− 3.44	9.80	22.60	0.86
SpikeSlab	0.75	− 0.30	5.10	10.90	0.91	0.81	0.24	6.56	15.12	0.91
GLM	0.99	0.35	1.22	2.62	1.00	0.35	− 6.68	15.39	35.49	0.73
SLR	0.66	− 0.30	5.90	12.60	0.90	0.72	0.26	7.19	16.59	0.89
KNN	0.72	− 0.36	5.93	12.66	0.85	0.79	− 0.41	6.34	14.62	0.92
XGB	0.97	0.02	1.87	4.00	0.99	0.66	1.70	8.38	19.31	0.83
Cubist	0.96	− 0.37	2.36	5.03	0.98	0.76	− 0.01	6.80	15.68	0.91

**Figure 6:**
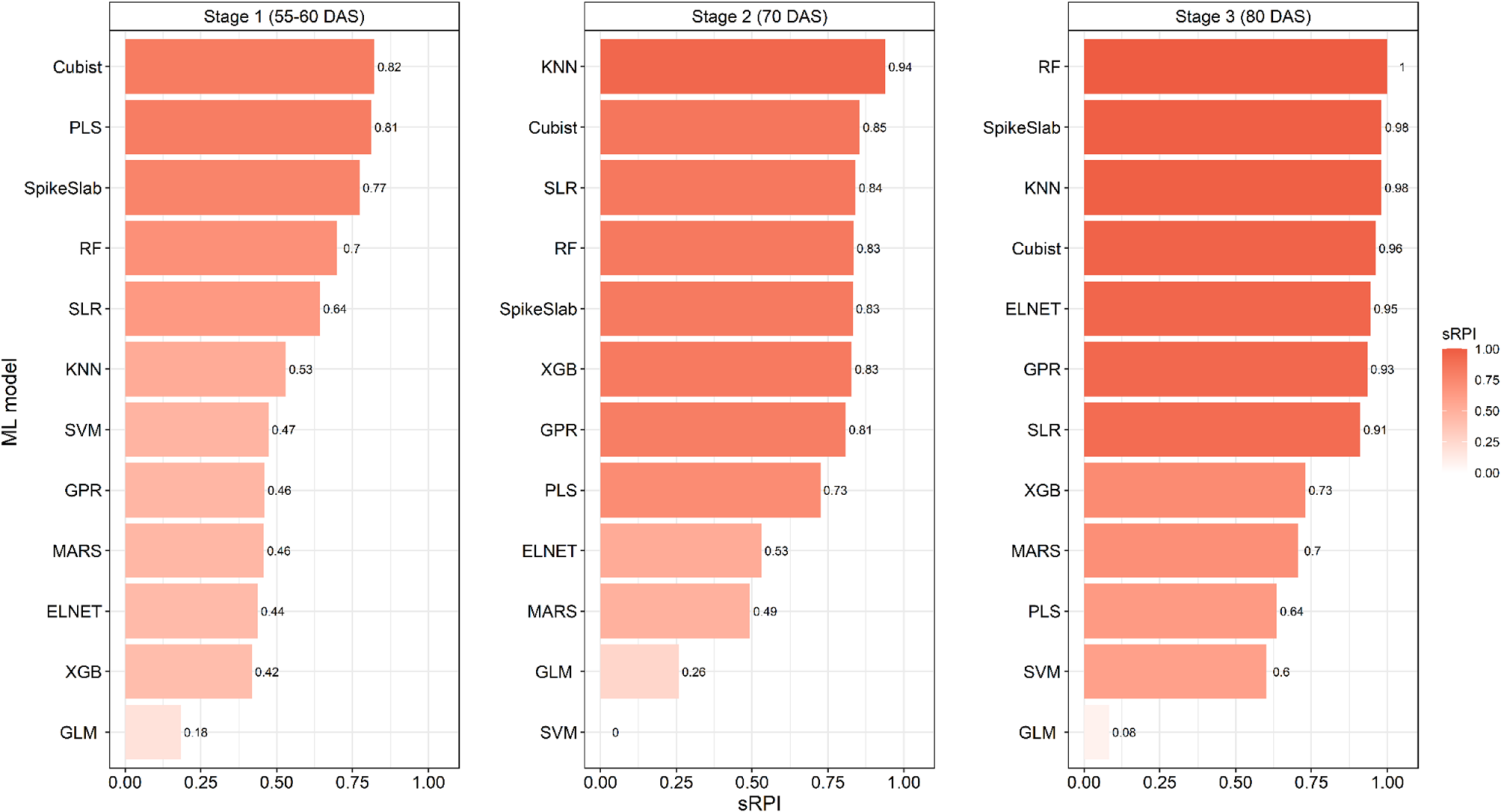
Stage wise** r**anking of ML models using the validation sRPI values for predicting wheat yield under different yellow rust severity level.

The validation output of different ML models in terms of the prediction of crop yields using the stage 3 data set is presented in Table 4. Based on the model prediction error indicator (n-RMSE), the RF, ELNT, GPR, GPR, MARS, SpikeSlab, SLR, KNN, XGB, and Cubist models are categorized under the good prediction class (10–20%). The RMSE of these models ranged from 5.31 to 8.51 q/ha and the model agreement index (d-index) ranged from 0.83 to 0.95. Further, the model GLM was found to be the poorest among all with the highest percent of model prediction error (n-RMSE) of 35.49% and RMSE of 15.39 q/ha. The models PLS and SVM were categorized as fair prediction class (n-RMSE between 20 and 30%). The detailed ranks of all the model at different stages using the validation sRPI values are presented in Fig. 6.

## Discussion

This research set out to investigate the effects of different intensities of wheat yellow rust on crop biophysical parameters, and to estimate yield through the integration of thermal and visual imaging with machine learning algorithms. Wheat yellow rust has a significant negative influences on crop growth and physiology, and these negative impacts are in direct proportion to the degree of rust severity. As the disease severity increased, the biophysical parameters such as photosynthesis rate, transpiration rate, stomatal conductance, LAI, MSI and RWC were significantly reduced. The discussion has been structured into subsections to improve the understanding of the findings.

### Photosynthesis, transpiration, stomatal conductance and intercellular CO_2_

Stressful environmental conditions have long been known to cause significant harm to the photosynthetic pigments of plants^48^. In wheat, the yellow rust fungus causes damage to the plant by growing in the leaves and producing spores that erupt through the leaf surface^49^, which reduces the effective green leaf area of the crop resulting in reduced photosynthesis rate in the plants. The reduced photosynthesis due to yellow rust is also attributed to the reduced chlorophyll content in the crop^50,51^. Abdulbagiyeva et al.^52^ also studied the effect of yellow rust in six bread wheat cultivars of Peninsula, they reported that the yellow rust resulted significant reduction in the photosynthesis rate, mainly due to reduction in the net assimilating area of the plant. The reduced stomatal conductance in wheat might be attributed to the damaged stomata due to the eruption of pathogen through the stomata^49^. According to findings of Smith et al.^53^, the transpiration rate of wheat affected by yellow rust of wheat decreased by 10%. Their study also noted an initial surge in the transpiration rate due to the disruption of the leaf epidermis by the rust pathogen, which hindered stomatal closure, however, as the disease progresses the effect is reversed owing to the decrease in effectively transpiring leaf area.

The intercellular CO_2_ concentration went up in the susceptible cultivars due to reduced stomatal conductance, which is slightly compensated by the reduced photosynthesis with disease progress, resulting higher or equivalent concentrations in some cases where no significant differences in adjacent categories cultivars were observed. However, numerically the intercellular CO_2_ concentration of the susceptible cultivars always remained significantly higher than the resistant cultivars. Mandal et al.^54^, in their research revealed that psyllium's downy mildew decreased plant photosynthesis and stomatal conductance, while increasing the intercellular CO_2_ concentration. Additionally, Zhao et al.^55^ also observed a similar increase in intercellular CO_2_ concentration in sugarcane leaves affected by orange rust.

### Canopy temperature

As per our findings, yellow rust disease severity is linked with an increased canopy temperature and a negative correlation with the transpiration rate. Smith et al.^53^ noted a temperature rise of 0.2–1.0 °C in foliage due to yellow rust lesions ten days after infection. This temperature increase was caused by rupturing of the epidermis, which prevented stomatal closure. Likewise, Wang et al.^56^ observed a similar trend in cucumber plants affected by wilt pathogen, where reduced stomatal conductance and transpiration led to an increase in temperature.

Our findings of temperature increase with reduced transpiration and stomatal conductance are supported by other researchers, such as Lindenthal et al.^57^ and Oerke et al.^58^. In addition, several studies have reported similar trends of higher canopy temperature after pathogen infection, which were used to identify diseases in various crops. For example, verticillium in Olive^59^, powdery mildew of tomato^60^, head blight of wheat^61^, downy mildew of grapes^62^ etc.

### LAI and NDVI

The LAI followed trend of decreasing values with increasing disease severity, which is due to the poor crop growth and canopy development due to yellow rust infection^51^. The LAI is key parameter linked with photosynthesis and transpiration rate of the canopy^63^ and as we have already mentioned the reduction of these basic physiological parameters of the crop due to the disease incidence, it was expected to observe LAI reduction with increasing disease severity. The reduced photosynthesis and green leaf area are responsible for poor and stunted growth of the crop. This reduces the leaf area per unit ground area which is captured in the LAI measurements, that showed a continuous decline with increasing levels of disease severity. The yellow rust reduces the green leaf area of the crop, induces chlorosis and necrosis. These changes in plants induces foliar shrinkage and senescence which results in poor LAI of the crop under diseased conditions^64^. The NDVI also showed a decreasing value as the disease severity increases. The NDVI measures the reflectance of the crop in NIR and Red wavelengths and the yellowing of crops tend to reduce the NDVI^65^. Su et al.^3^ discovered that the yellow rust of wheat increases the Red and decreases the NIR reflectance, due to chlorophyll reduction, which makes NDVI as a useful index for monitoring yellow rust in wheat. Several researchers have reported reduced NDVI due to yellow rust and used it for monitoring rust field using aerial images of the field^20,66^.

### MSI and RWC

The MSI is linked with cell membrane damage due to stress that results in change in membrane permeability and leakage of electrolytes^67^. Recently, researchers have reported increased electrolyte leakage due to yellow rust in wheat cultivars, which might have resulted in low MSI of the crops with increasing disease severity^51^. The wheat yellow rust pathogen directly damages the cell while erupting from the leaf to the surface, which might have attributed to low MSI of wheat cultivars. Sabir et al.^68^ also reported reduced MSI of wheat cultivars due to yellow rust. The RWC showed decreasing trends as the disease severity increases. Chen et al.^51^ in their study reported a lower value of RWC in yellow rust susceptible cultivar as compared to the resistant wheat cultivars, however the differences were not statistically significant in their study.

### Crop yield

The crop yield was significantly reduced with increasing disease severity. The yield reduction is attributed to several factors. The reduced green leaf area has reduced the total photosynthesis, light interception as well as its use efficiency, which is one of the primary reasons behind the reduced crop yield^49^. The yellow rust on wheat reduces the sugar supply to the developing seed resulting in smaller seeds. The flag leaf and the upper second leaf are considered most important for producing sugars for the developing grain and as soon as these are affected from the rust, the yield of wheat crop falls substantially^69^. The yield loss due to the yellow rust infection is attributed to the reduced kernels per spike and low-test weight^70^, which is due to the serious destruction of the photosynthetic function of the leaves affecting the assimilation of the products^71^. Smith et al.^53^ reported a yield reduction of 46–51% due to yellow rust of wheat. Park et al.^72^ reported a yield loss of 15–25% in plants with some level of resistance to yellow rust, whereas in susceptible cultivars the yield loss reported was 45–50%. Jindal et al.^73^ assessed the losses due to yellow rust in some common wheat varieties of India, out of  which few are also part of our study, and reported a loss ranging from 4.2–68.3%. All the above findings on wheat yield loss due to yellow rust is in line with our findings.

### Yield prediction using ML models

The ML models used in our study were able to make good predictions of yield under yellow rust affected conditions using the image indices and biophysical parameters as training dataset.

As evident from our results, following the rust infections, cell membranes become more damaged, resulting in water loss, causing dehydration, and wilting in plants. This situation also leads to the closure of stomata, altering heat loss from the leaf surface and affecting leaf surface temperature. When crop diseases begin, the shifts in heat radiation due to increased plant water loss, stomata closure, and heightened respiration can be observed in thermal image derived canopy temperature. These alterations along with changes in plant pigmentation due to yellow rust^49,74,75^ were effectively captured by RGB and thermal image indices. The variations in the values of these indices were utilized to quantify crop yield through machine learning models. In comparison to similar studies, our results show better values for R^2^, d-index, RMSE, n-RMSE and MBE. This may be due to the inclusion of biophysical parameters of the crop that are strongly correlated with plant health. We also observed that, while prediction accuracy improves as we move closer to harvesting, the initial predictions made during first stage are more crucial for decision making, despite having acceptable but lower accuracies, compared to the predictions made closer to harvesting. The validation output of different models indicated that Cubist, PLS, and SpikeSlab models were found to be the effective models to predict the wheat yield at an early stage of the crop. The KNN, Cubist, SLR, RF, SpikeSlab, XGB, GPR and PLS models proved to be more useful in predicting crop yield at middle stage of the crop. Further, this research also demonstrated that at late stage of the crop, the RF, SpikeSlab, KNN, Cubist, ELNET, GPR, SLR, XGB and MARS models were found good to predict the crop yield. As there are no comparable studies to ours, we have instead compared our research to similar work on yield prediction that used machine learning. Ruan et al.^26^ recently employed several ML algorithms, such as ELNET, SVM, KNN, RF, and XGB, in combination with proximal sensing and weather data to predict wheat yield. Their findings indicate that RF and XGB were the top two models for predicting wheat yield, with R^2^ of over 0.70 and RMSE ranging from 0.75–0.85 t/ha. Li et al.^76^ used remote sensing-based vegetation indices to predict wheat yield in China with reasonable accuracy using RF and SVM. Han et al.^77^ tested KNN, SVM, GPR, ANN and DT (decision tree) methods for predicting wheat yield in China. Their results suggested that the models were able to predict yield with good accuracy with R^2^ > 0.75 and n-RMSE value less than 10%. SVM, GPR and RF were top three models for yield prediction in their study. Fei et al.^78^ used UAV based multi-sensor data to predict wheat yield using ML algorithms. They tested Cubist, SVM, DNN, ridge regression and RF methods with RGB, multispectral and thermal camera data. Results of their study revealed that the model performance improves using muti-sensor data as compared to the single sensor. Using single sensor data, they achieved R^2^ values of 0.527–0.670, while in the ensemble approach the R^2^ up to 0.692 were observed. This falls in line with results of our study, though we do not test the single sensor approach, however from the literature we observed that using the thermal and visible images with actual biophysical parameters of the crop improved the prediction accuracy considerably. Kang et al.^79^ tested the Lasso, SVM, RF XGB and CNN models to predict maize yield in United States. They found that the XGB outperforms all the models in terms of accuracy and stability. Going forward, the prediction of yield could be expanded to include satellite-level data, and the inclusion of additional agroclimatic and soil variables could enhance the accuracy of the results by utilizing machine learning and deep learning models.

## Conclusions

This study investigated the influence of yellow rust on the biophysical parameters of wheat cultivars with varying degrees of rust resistance. Using thermal and RGB image indices in combination with biophysical parameters, ML models were constructed to estimate yield under diseased conditions. The biophysical parameters, including photosynthesis rate, transpiration rate, stomatal conductance, LAI, MSI and RWC were dropped significantly due to rust, and the reductions were directly correlated with levels of rust severity. The yield reduction in moderate resistant, low resistant and susceptible cultivars as compared to resistant cultivars, varied from 15.9 to 16.9%, 28.6 to 34.4% and 59.0 to 61.1%, respectively. The ML models were able to predict the yield of rust-affected wheat crop before harvest with reasonable accuracy. The Cubist, PLS, and SpikeSlab models were found to be effective models for predicting the wheat yield at an early stage of crop growth (55–60 DAS). The KNN, Cubist, SLR, RF, SpikeSlab, XGB, GPR and PLS models were proved to be more useful in predicting crop yield at middle stage of crop growth (70 DAS) and at late stage of crop growth (80 DAS) RF, SpikeSlab, KNN, Cubist, ELNET, GPR, SLR, XGB and MARS models were found good to predict the crop yield. This study quantifies the changes in biophysical parameters in wheat cultivars with different rust resistant levels and suggests the potential of ML models developed using biophysical parameters and image-based indices to assess the crop yield, which can be used for sustainable management of resources.

### Supplementary Information

Supplementary Figures.

Supplementary Tables.

## Data Availability

Data supporting the findings of this study are available from the corresponding author on reasonable request.
