# Supplementary material for: Combining biophysical parameters with thermal and RGB indices using machine learning models for predicting yield in yellow rust affected wheat crop
Source: Sci Rep. 2023 Nov 1;13:18814. doi: 10.1038/s41598-023-45682-3 (PMC10620169; doi:10.1038/s41598-023-45682-3)
Supplement: Supplementary file 1 — Supplementary Figures. [file 41598_2023_45682_MOESM1_ESM.docx]

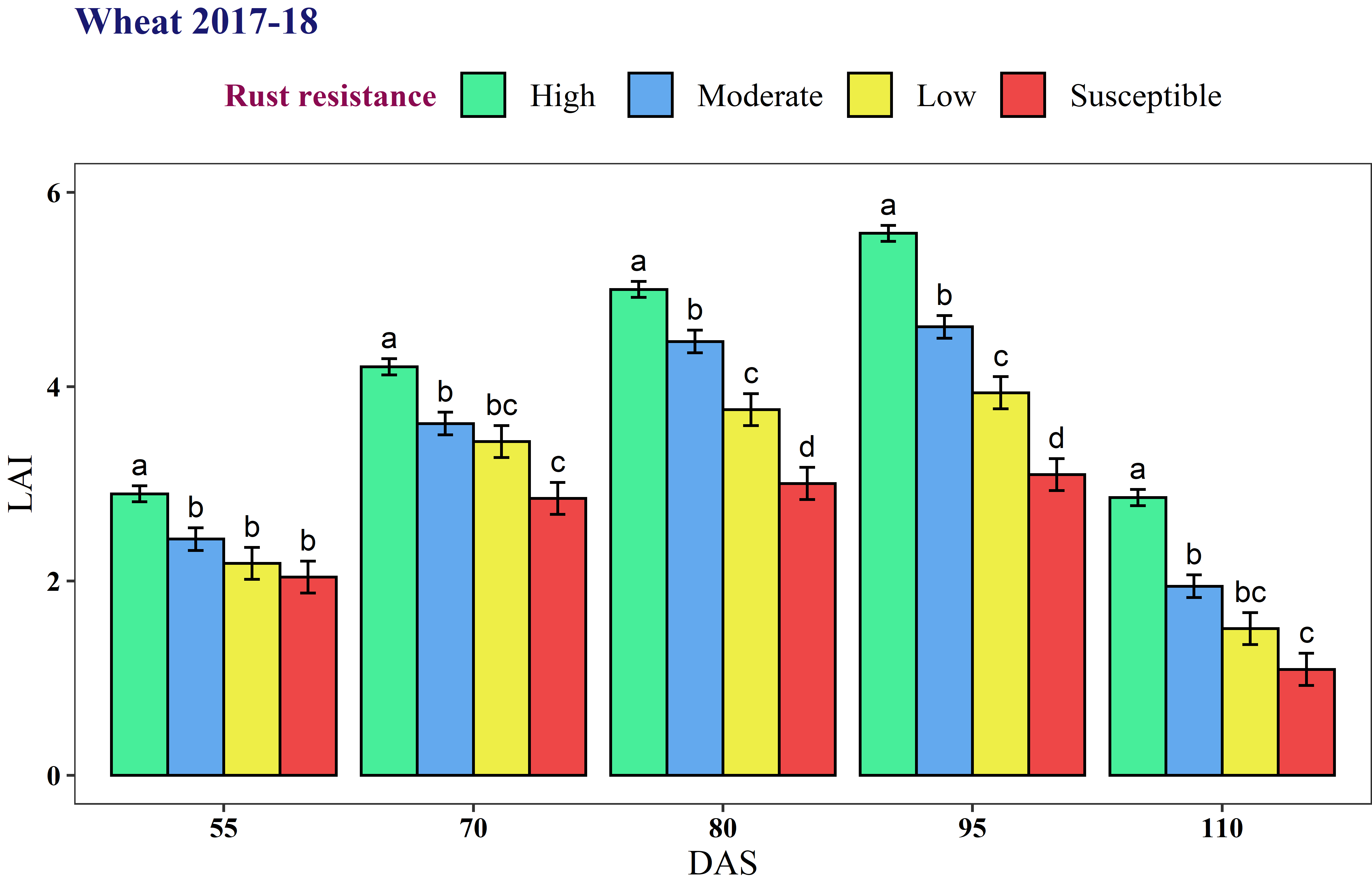

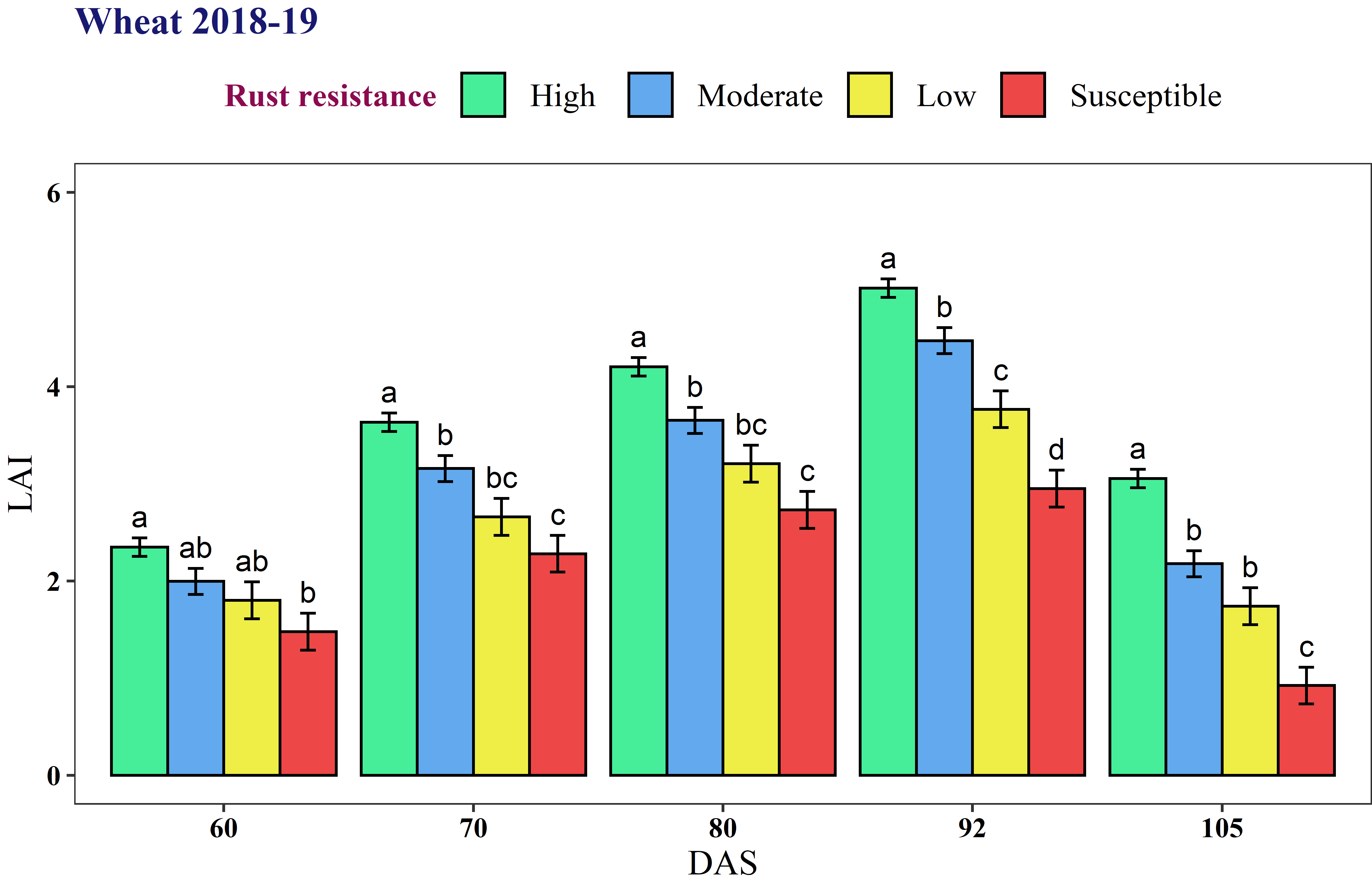


**Supplementary Fig. 1 :** LAI variations under different levels of wheat yellow rust severity.


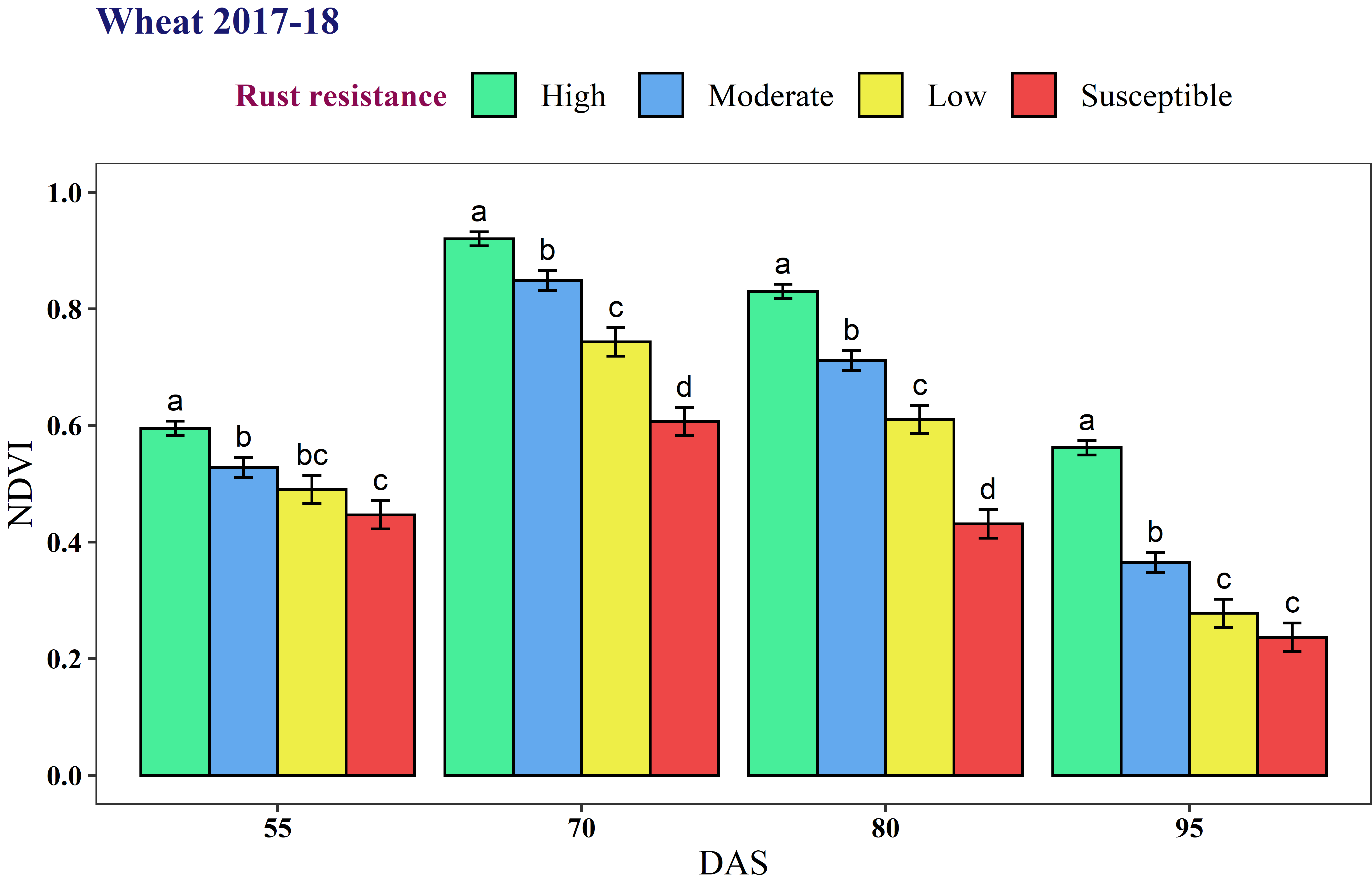

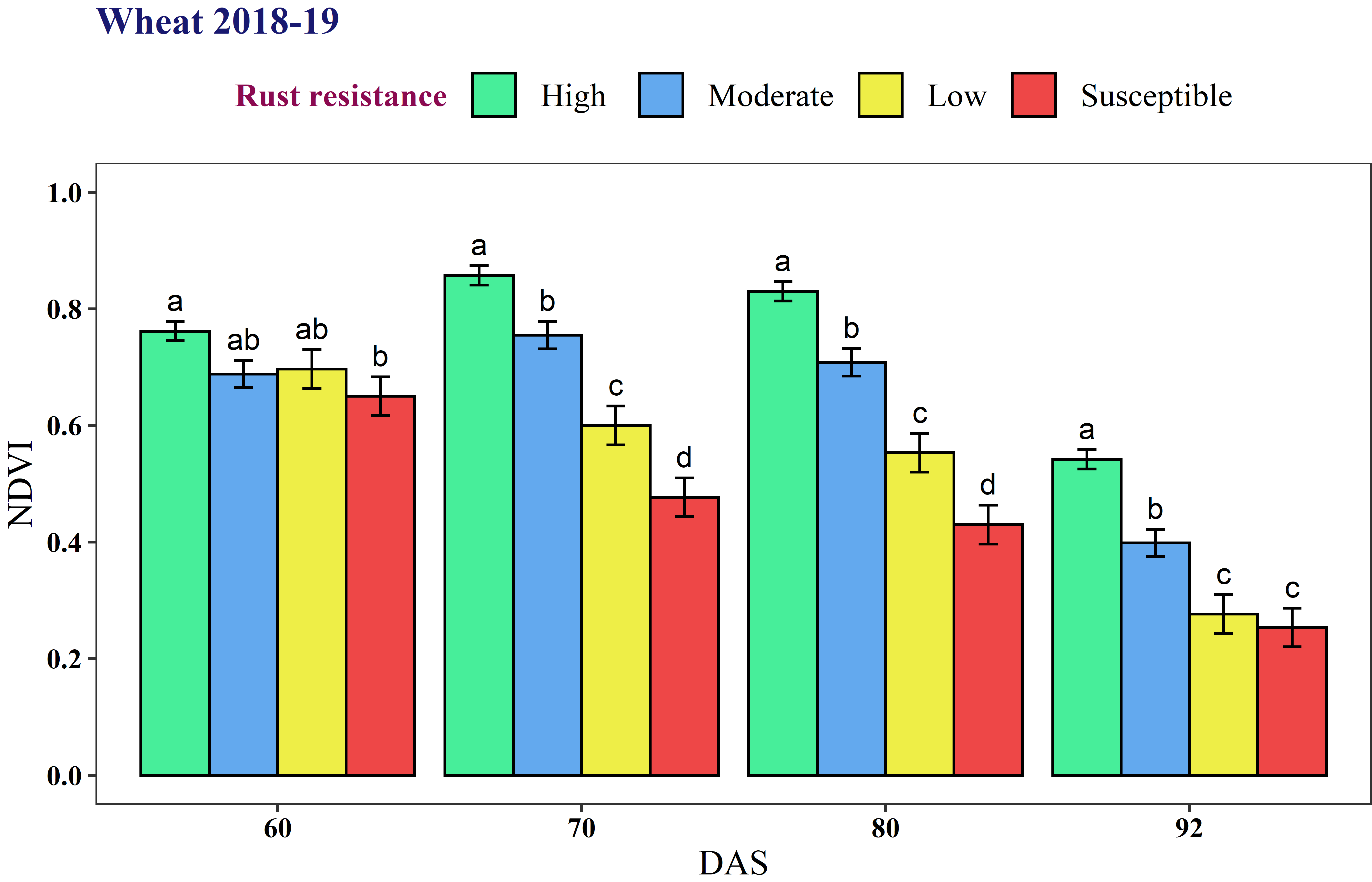


**Supplementary Fig. 2.** NDVI variations under different levels of wheat yellow rust severity


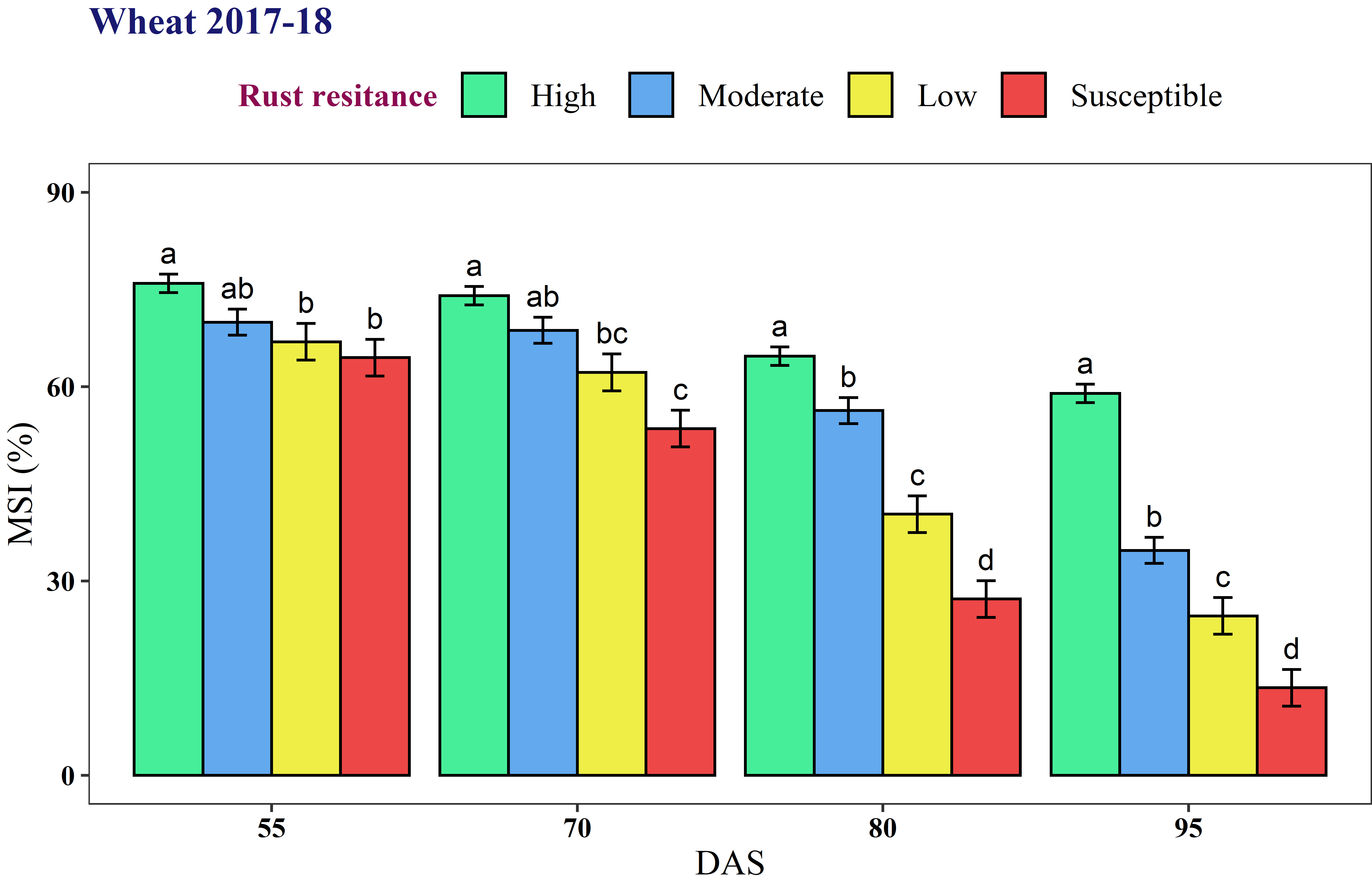

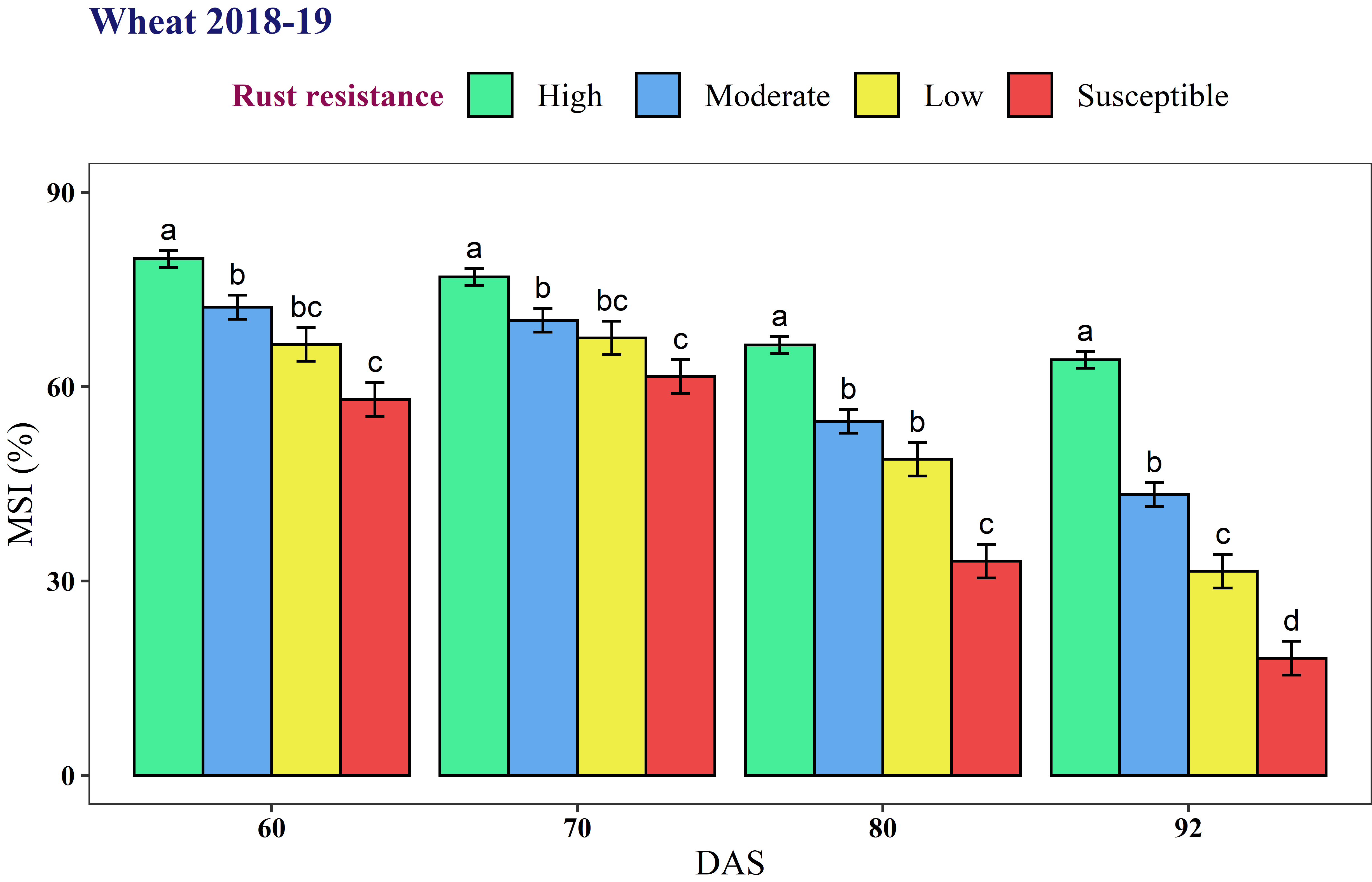


**Supplementary Fig. 3:** MSI variations under different levels of wheat yellow rust severity


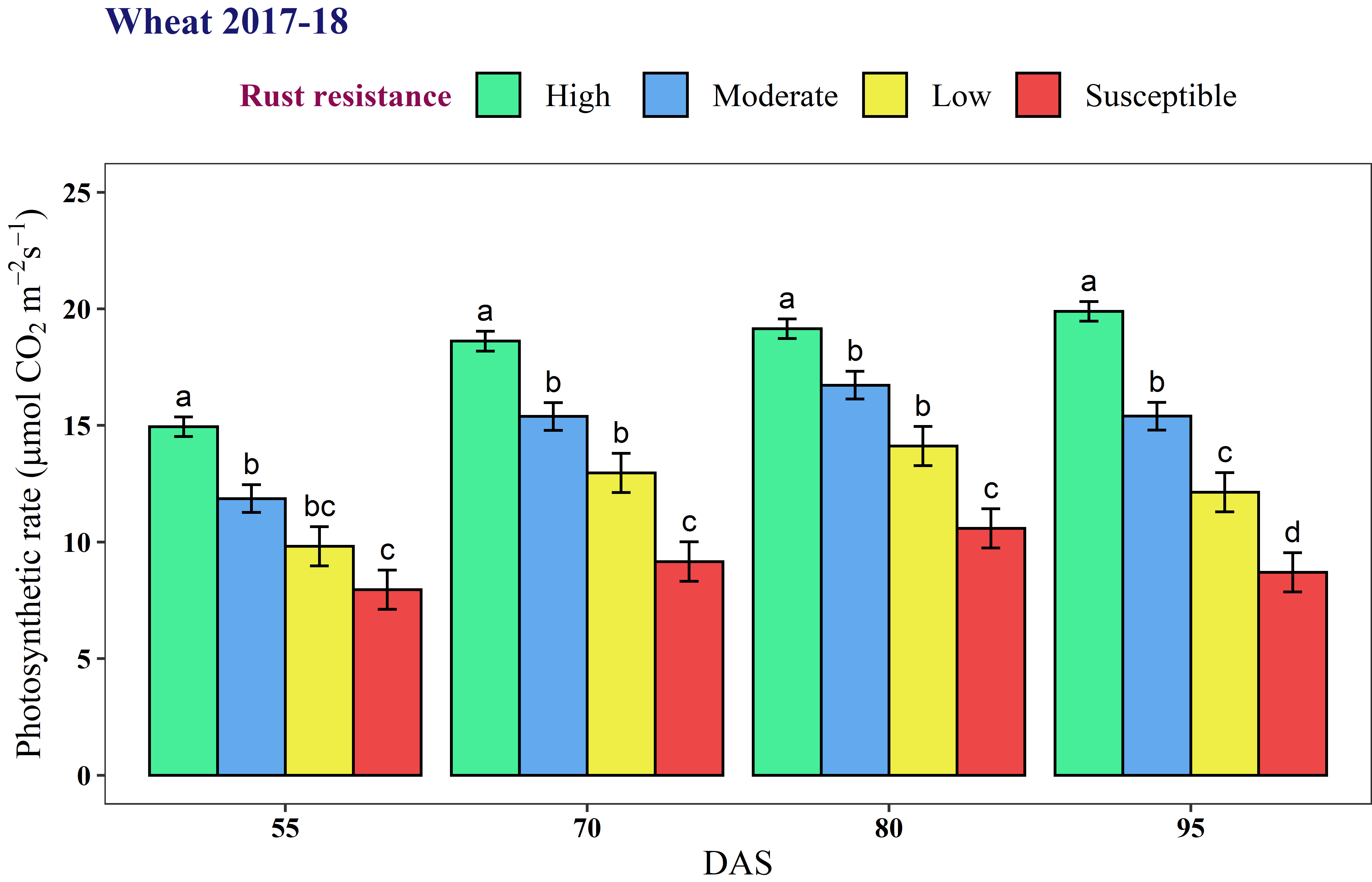

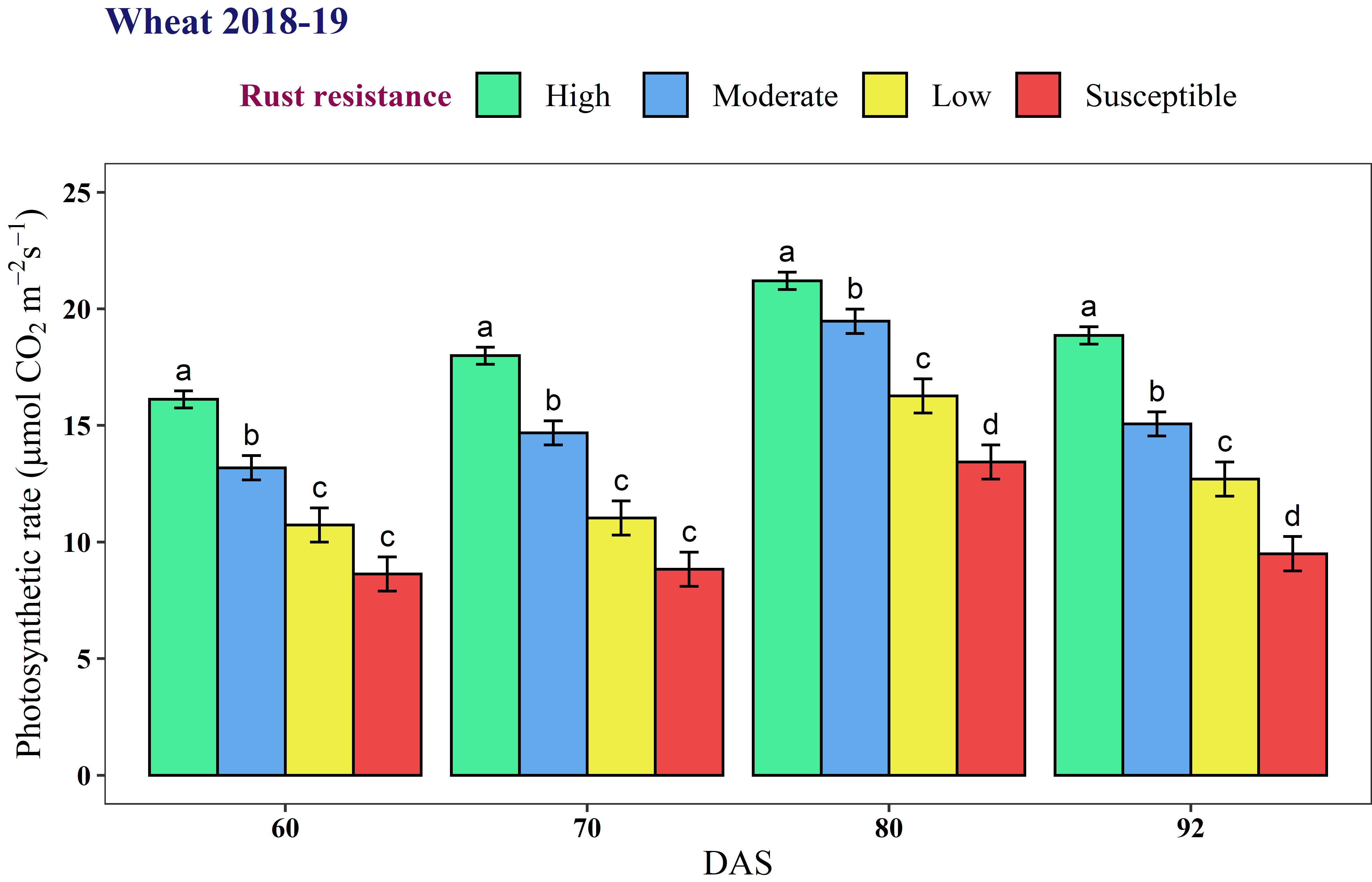


**Supplementary Fig. 4 :** Photosynthesis rate variations under different levels of wheat yellow rust severity


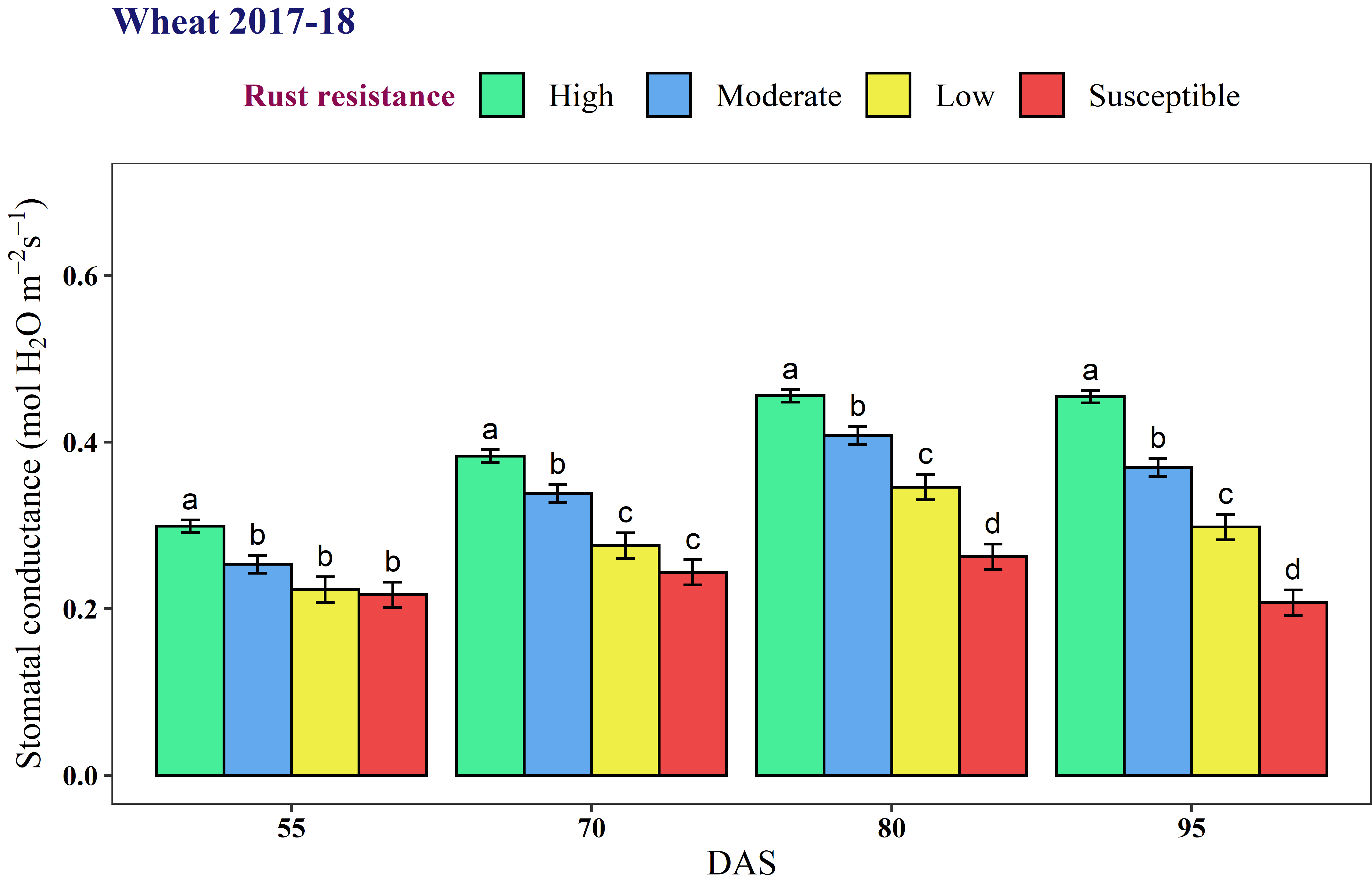

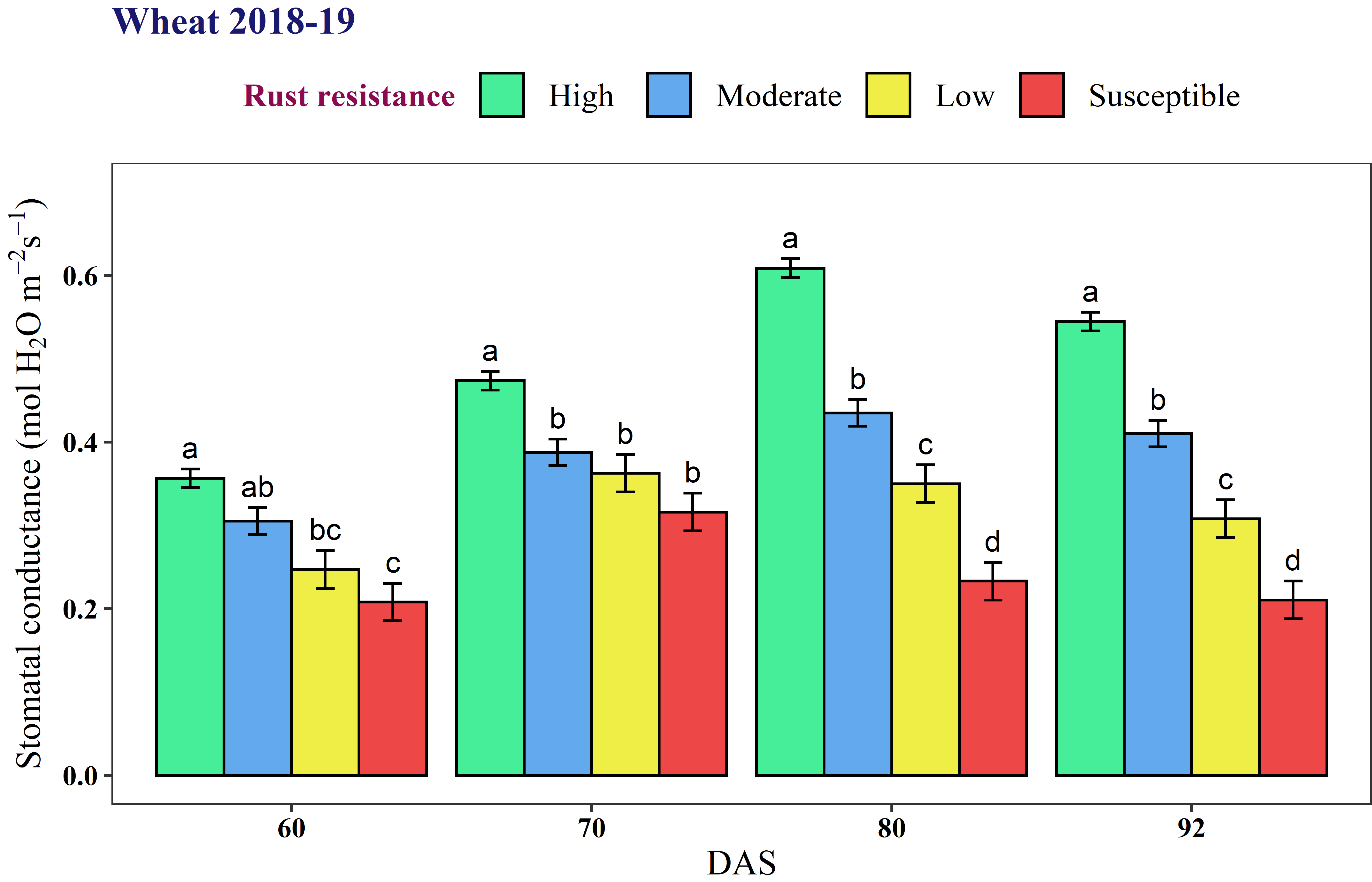


**Supplementary Fig. 5 :** Stomatal conductance variations under different levels of wheat yellow rust severity


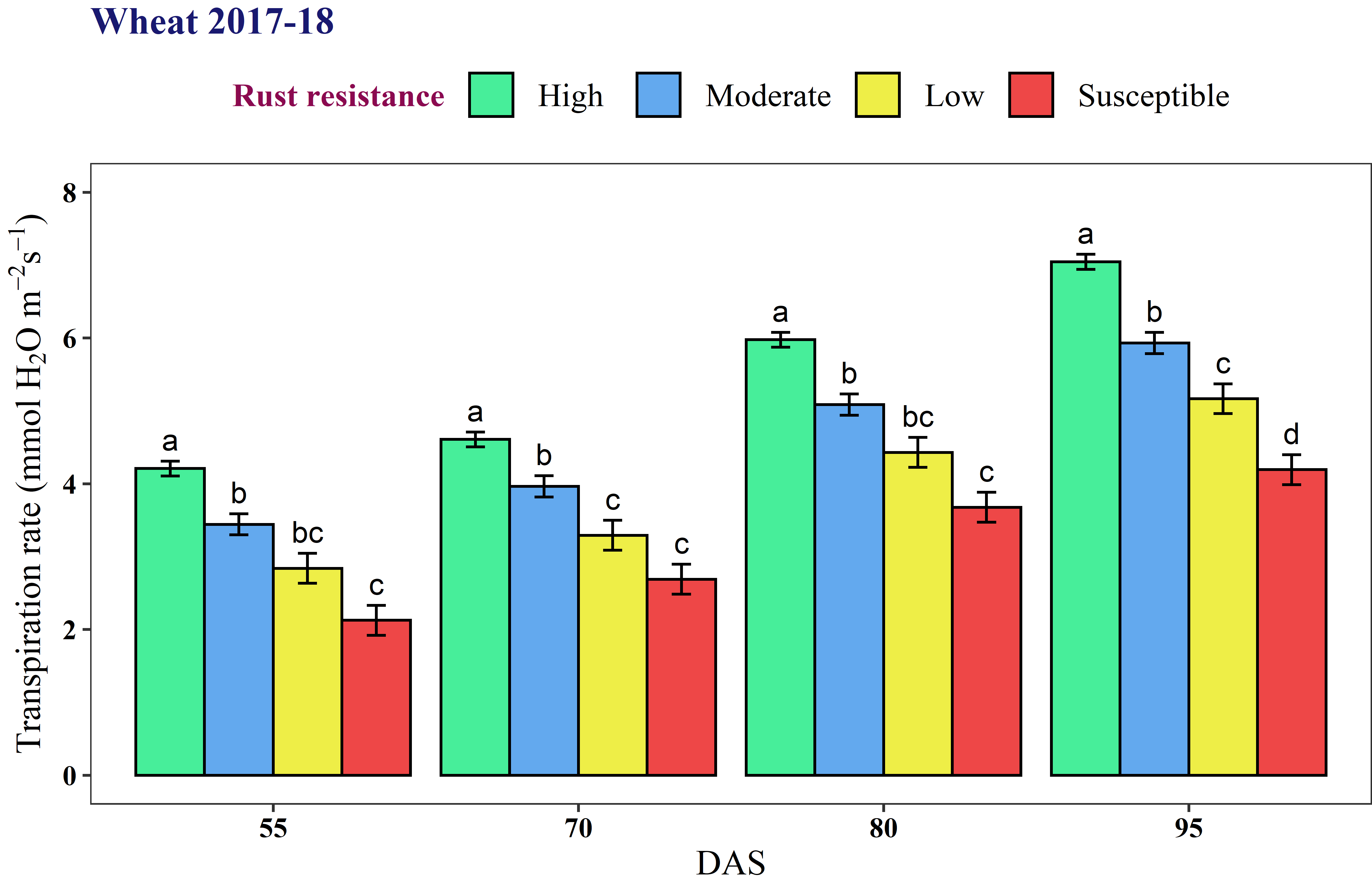

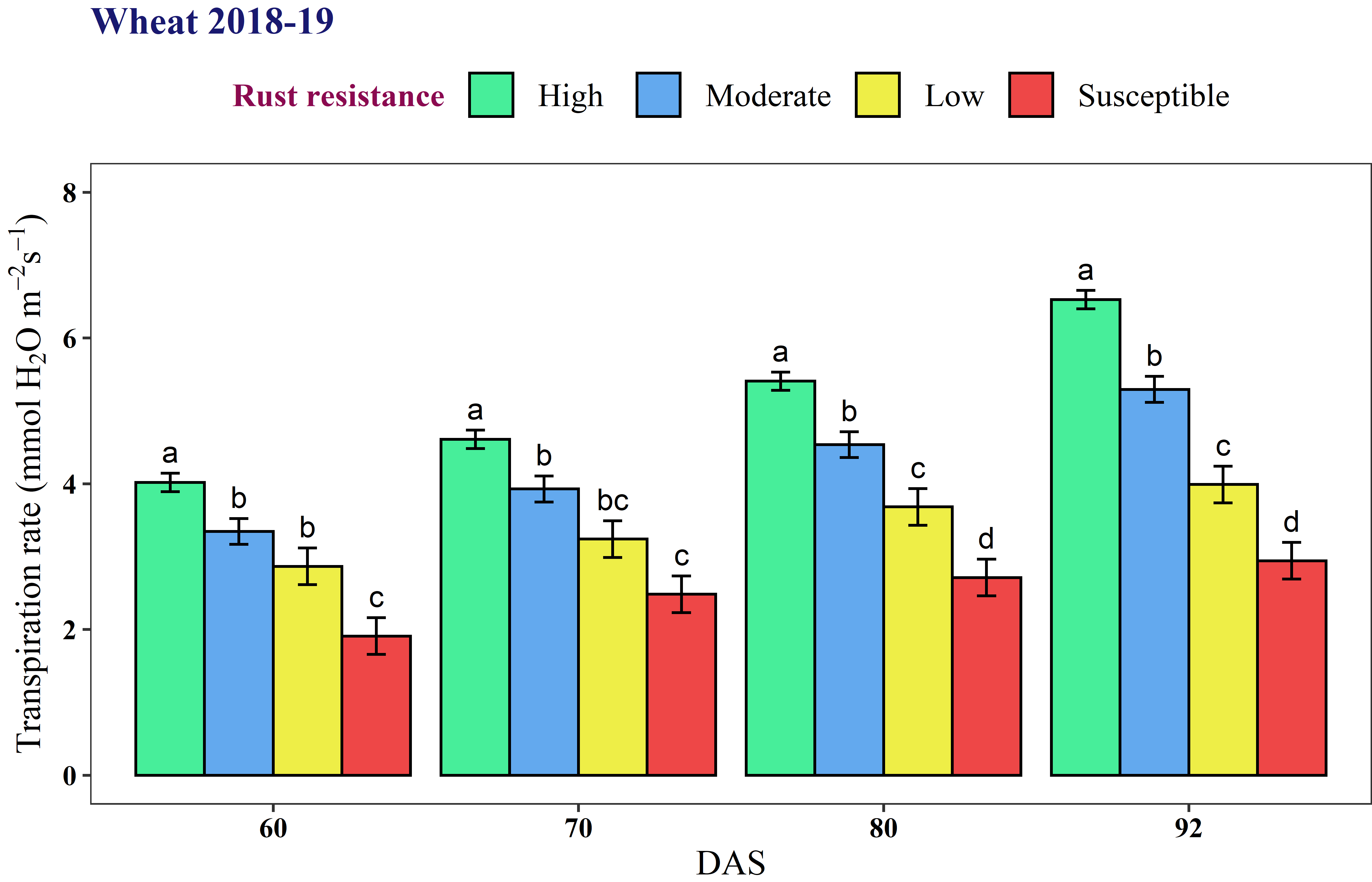


**Supplementary Fig. 6 :** Transpiration rate variations under different levels of wheat yellow rust severity


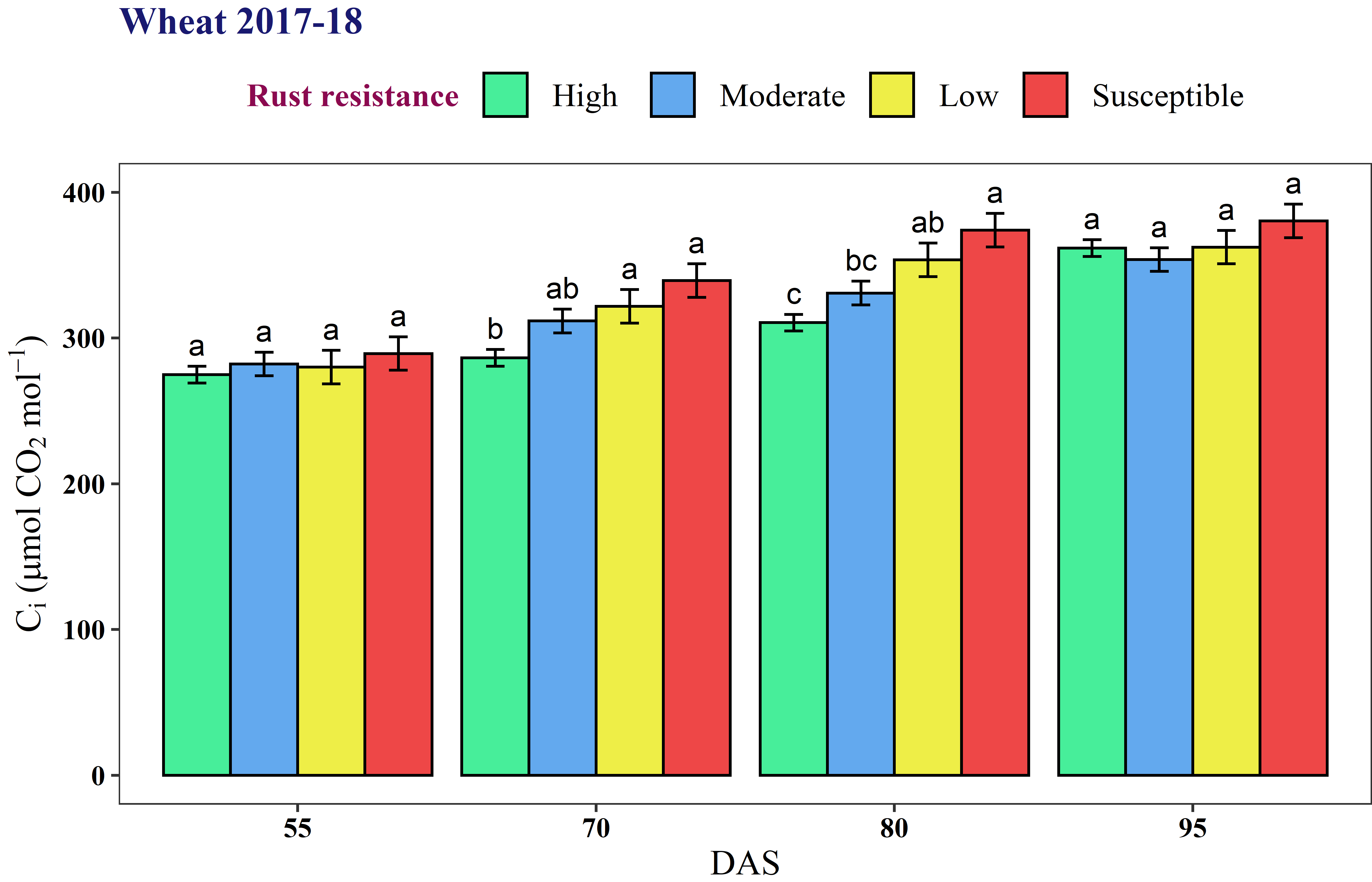

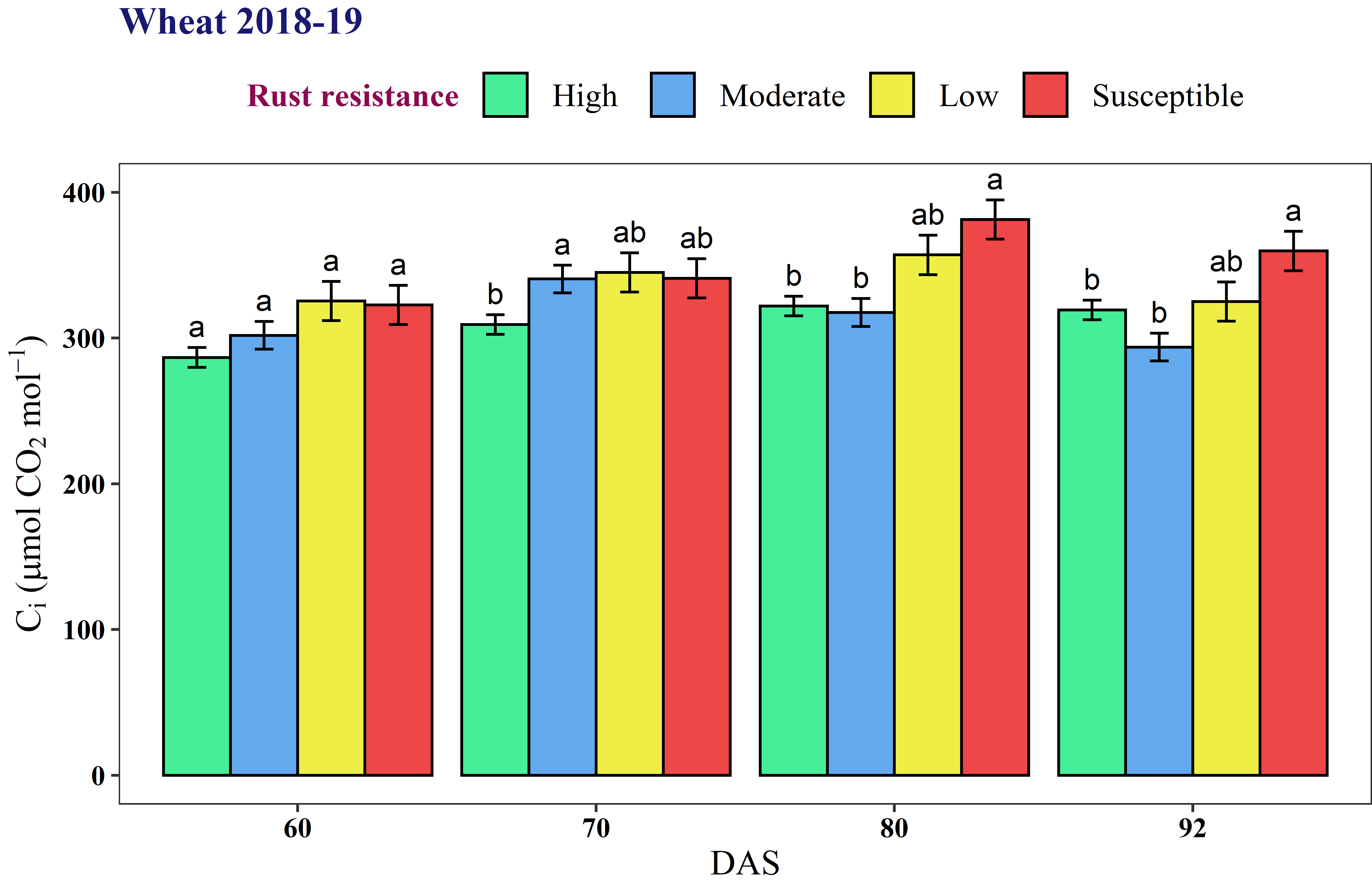


**Supplementary Fig. 7:** Intercellular CO_2_ concentration variations under different levels of wheat yellow rust severity


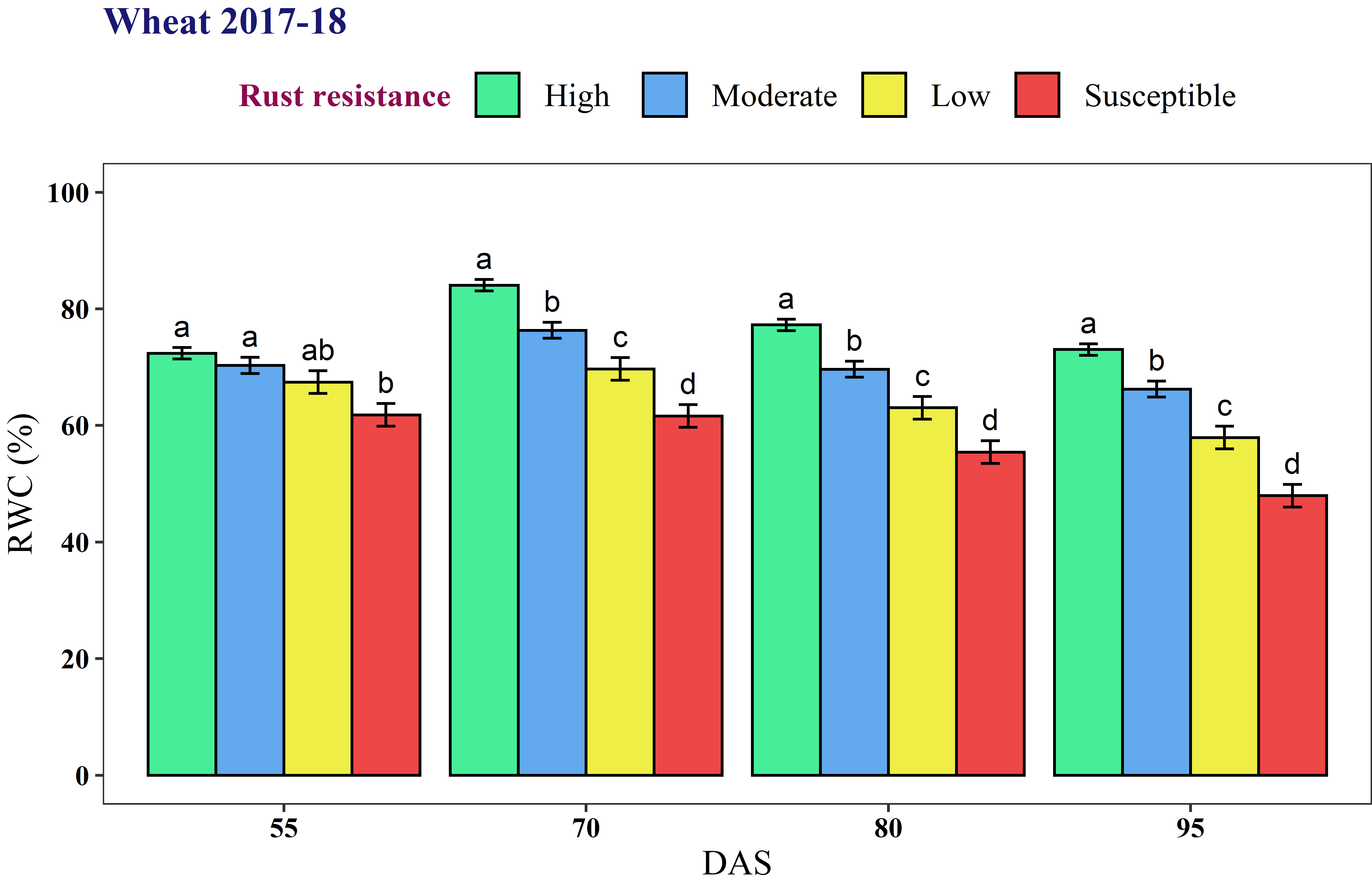

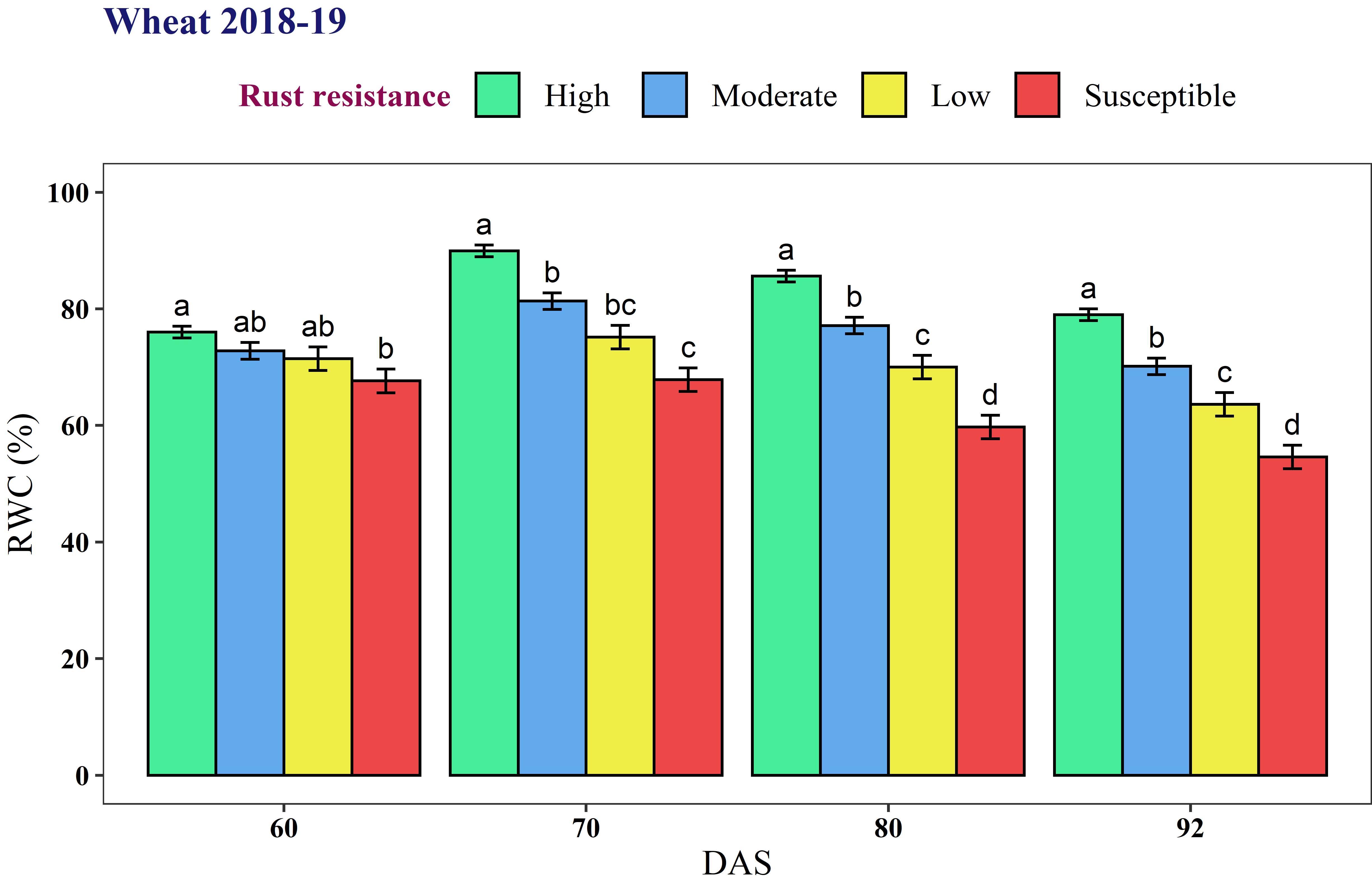


**Supplementary Fig. 8 :** RWC variations under different levels of wheat yellow rust severity


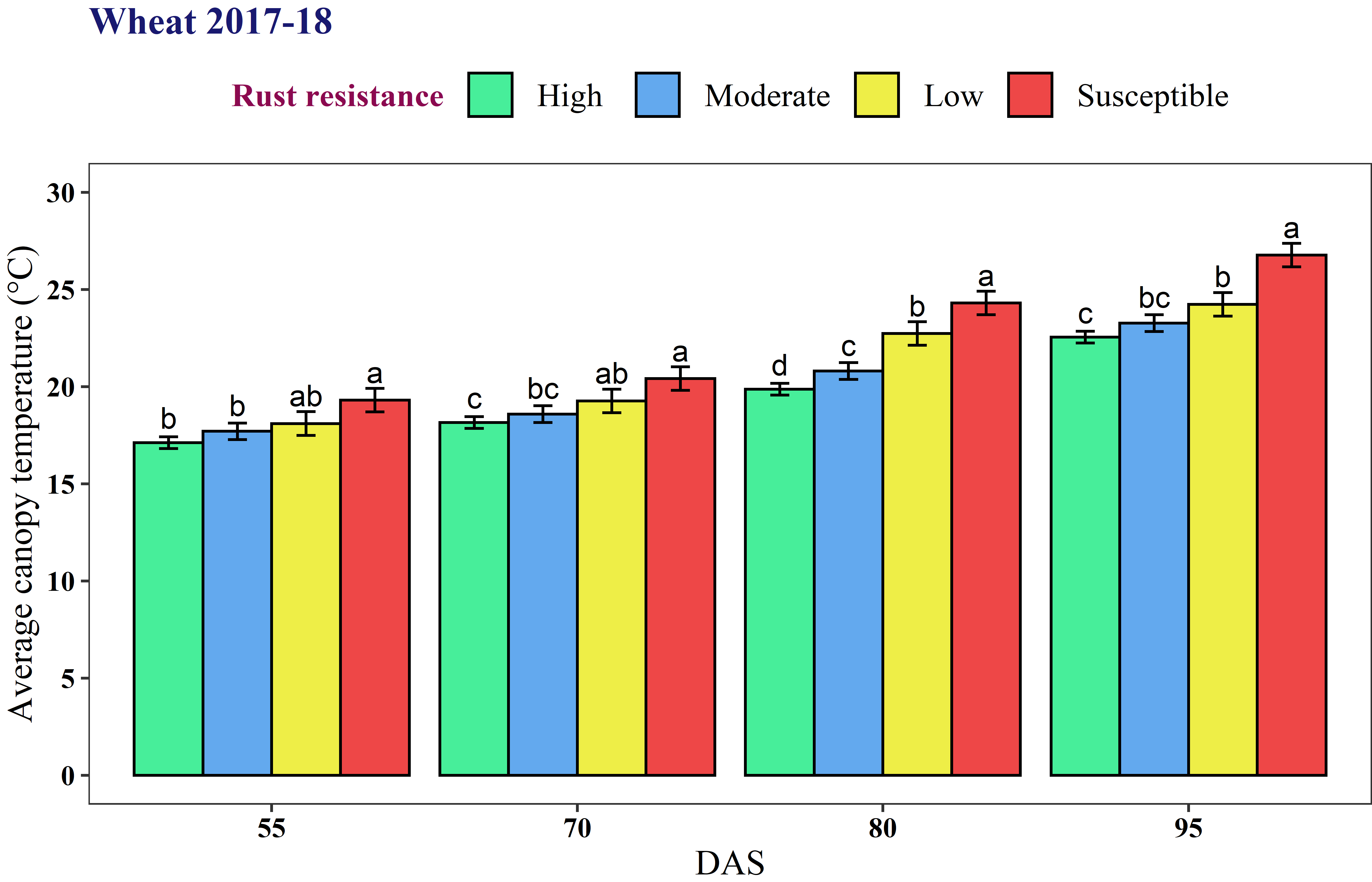

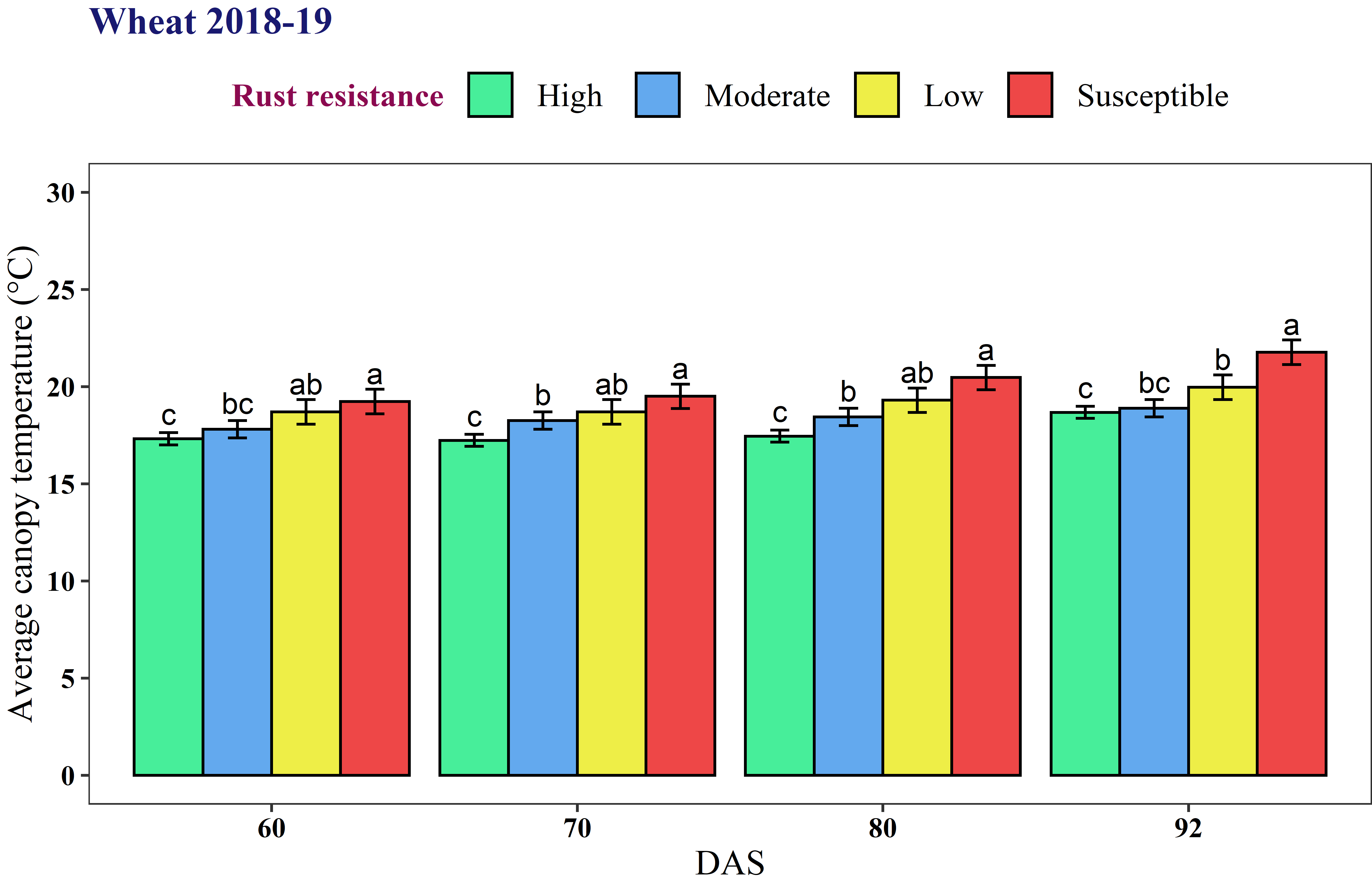


**Supplementary Fig. 9:** Average canopy temperature variations under different levels of wheat yellow rust severity


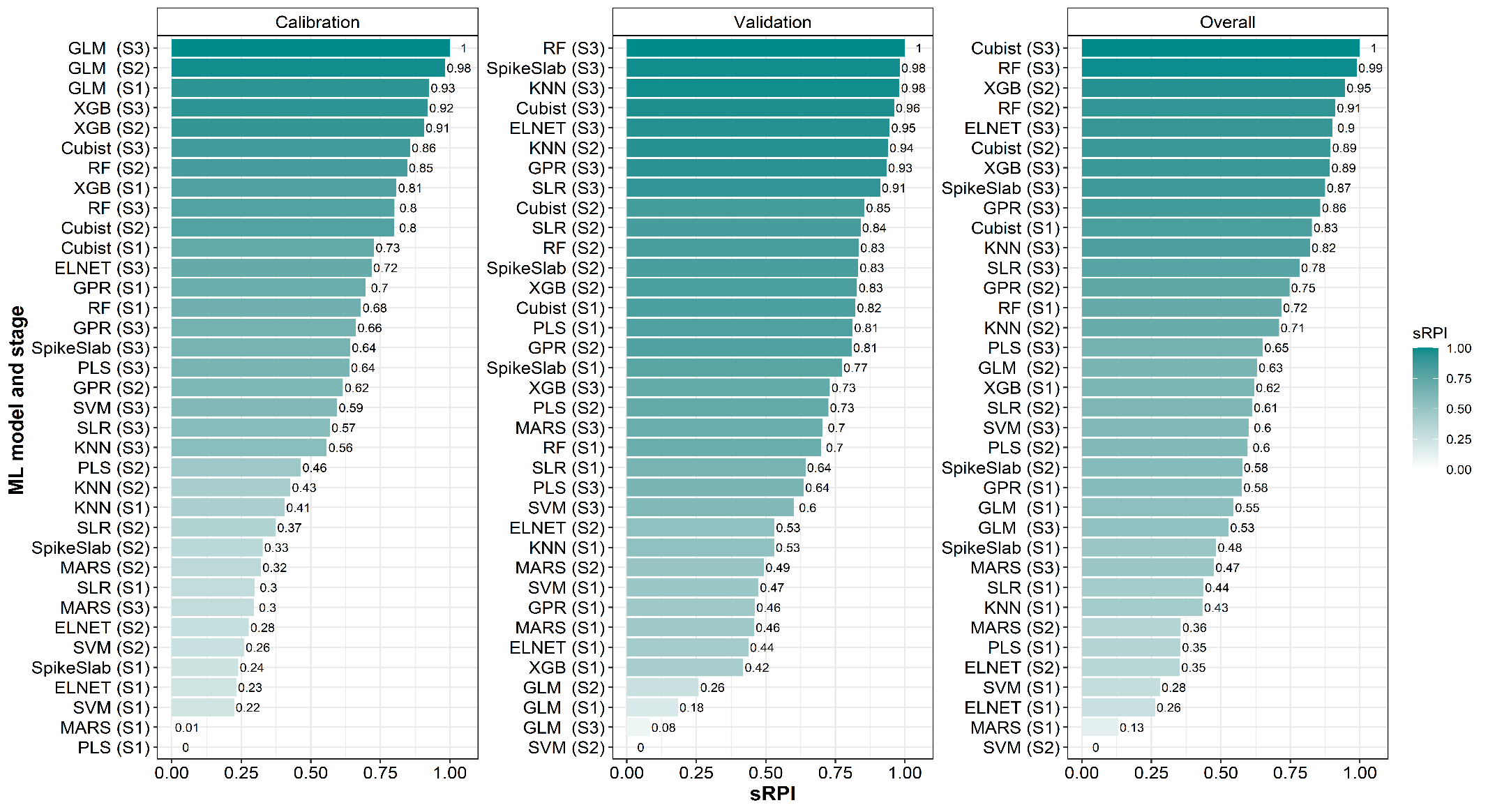


**Supplementary Fig. 10** : Ranking of all ML models and stage using the sRPI values for predicting wheat yield under different levels of yellow rust severity. *S1, S2 and S3 represents Stage 1, Stage 2 and Stage 3 of the yield prediction, respectively*
