# Supplementary material for: Combining biophysical parameters with thermal and RGB indices using machine learning models for predicting yield in yellow rust affected wheat crop
Source: Sci Rep. 2023 Nov 1;13:18814. doi: 10.1038/s41598-023-45682-3 (PMC10620169; doi:10.1038/s41598-023-45682-3)
Supplement: Supplementary file 2 — Supplementary Tables. [file 41598_2023_45682_MOESM2_ESM.docx]

**Supplementary Table 1:** Details of visible and thermal indices used in the study.

| S.No. | Name | Abbreviation | Definition | References |
| --- | --- | --- | --- | --- |
| ***Visible indices*** | | | | |
|  | Red | R | Red (0-255) | - |
|  | Green | G | Green (0-255) | - |
|  | Blue | B | Blue (0-255) | - |
|  | Normalised Red | r | $\text{R/(R+G+B)}$ | Xu et al. 2010 |
|  | Normalised green | g | $\text{G/(R+G+B)}$ | Xu et al. 2010 |
|  | Normalised blue | b | $\text{B/(R+G+B)}$ | Xu et al. 2010 |
|  | Colour intensity index | INT | $\text{(R + G + B)/3}$ | Ahmad and Reid, 1996 |
|  | Green red difference | GRD | $\text{G}\text{-R}$ | - |
|  | Blue red difference | BRD | $\text{B}\text{-R}$ | - |
|  | Green blue difference | GBD | $\text{G-B}$ | Xu et al. 2010 |
|  | Green red ratio index | GRRI | $\text{G/R}$ | - |
|  | Green blue ratio index | GBRI | $\text{G/B}$ | - |
|  | Red blue ratio index | RBRI | $\text{R/B}$ | - |
|  | Woebbecke index | WI | $\text{(G-B)/(R-G)}$ | Woebbecke et al. 1995 |
|  | Green-red vegetation index | GRVI | $\text{(G-R)/(G+R)}$ | Hunt et al. 2005 |
|  | Kawashima index | IKAW | $\text{(R-B)/(R+B)}$ | Kawashima and Nakatani, 1998 |
|  | Normalized Difference Turbidity Index | NDTI | $\text{(R-G)/(R+G)}$ | Lacaux et al. 2007 |
|  |  | GBI | $\text{(G-B)/(G+B)}$ | - |
|  |  | NGRDI | $\text{(}\text{G-R}\text{)/(R+G+B)}$ | - |
|  |  | NBRDI | $\text{(}\text{B-R}\text{)/(R+G+B)}$ | - |
|  |  | NGBDI | $\text{(G-B)/(R+G+B)}$ | - |
|  | Green leaf index | GLI | $\text{(2g}\text{-}\text{b}\text{-}\text{r)/(2g-b-r)}$ | Louhaichi et al. 2001 |
|  | Visible atmospherically resistant index | VARI | $\text{(g}\text{-}\text{r)/(}\text{g+r-b}\text{)}$ | Gitelson et al. 2002 |
|  | Normalized difference index | NDI | $\text{(g}\text{-}\text{r)/(}\text{g+r}\text{)}$ | Woebbecke et al. 1995 |
|  | Gray | Gray | $0\text{.2898×r + 0.5870×g + 0.1140×b}$ | Kazmi et al. 2015 |
|  | Brightness index | BI | $\sqrt{{{(R}^{2}{+G}^{2}+B^{2})}/3}$ | Levin et al, 2005 |
|  | Hue index | HI | $\text{(2×R-G-B)/(G-B)}$ | Levin et al. 2005 |
|  | Redness index | RI | $R^{2}\text{/(B×}G^{3}\text{)}$ | Levin et al. 2005 |
|  | Saturation index | SI | $\text{2 × (R-G -B)/(G - B)}$ | Levin et al. 2005 |
|  | Colouration index | CI | $\text{(R - B) / R}$ | Levin et al., 2005 |
|  | Color index of vegetation | CIVE | $\text{0.441×R - 0.811×G + 0.385×B + 18.78745}$ | Kataoka et al. 2003 |
|  | Vegetative Index | VEG | $\text{G/(}R^{0.667}B^{\text{0.334}}\text{)}$ | Hague et al. 2006 |
|  | Excessive green index | ExG | $\text{2g-r-b}$ | Meyer et al. 1999 |
|  | Excessive red | ExR | 1.4r-g | Meyer et al. 1999 |
|  | Excessive green index  minus excess red index | ExGR | 3g-2.4r-b | Meyer et al. 2004 |
|  | Modified Excess Green Index | MxEG | $\text{1.262G-0.884R-0.311B}$ | Burgos-Artizzu et al. 2011 |
|  | Excessive blue | ExB | 1.4×b-g | Wenhua Mao et al. 2003 |
|  | Principal component analysis index | IPCA | $\text{0.994 (R - B) + 0.961 (G-B) + 0.914 (G-R)}$ | Saberioon et al. 2014 |
|  | Red green blue VI | RGBVI | $\text{(}G^{2}\text{- (R×B))/(}G^{2}\text{+ (R×B))}$ | Bending et al 2015 |
|  |  | GLAI | $\text{(25 × (G - R)/(G +R - B) + 1.25 )}$ | - |
|  | Overall Saturation Index | SAT | $\text{(max(R,G,B) - min(R,G,B)) / max(R,G,B)}$ | - |
|  |  | OHI | $\text{atan}\text{(2 × (R - G - B) / 30.5 × (G - B))}$ | - |
|  | True Color Vegetation Index | TCVI | 1.4×(2R-2B)/(2R- G-2B+255×0.4) | - |
|  | Combined Indices 1 | COM1 | $\text{ExG}\text{ + CIVE + }\text{ExGR}\text{ + VEG}$ | Guijarro et al. 2011 |
|  | Combined Indices 2 | COM2 | $\text{0.36×ExG+ 0.47×CIVE + 0.17×VEG}$ | Guerrero et al. 2012 |
| ***Thermal indices*** | | | | |
|  | Crop water stress index | CWSI | ${\text{(T}_{\text{canopy}}\text{ -}\text{T}_{\text{wet}}\text{) }}/{\text{(T}_{\text{dry}}\text{-}\text{T}_{\text{wet}}})$ | Banerjee et al. 2020 |
|  | Stomatal conductance index | IG | ${{\text{(}\text{(T}_{\text{dry}}\text{-T}}_{\text{canopy}}\text{) }}/{\text{(T}_{\text{canopy}}\text{-}\text{T}_{\text{wet}}})$ | Banerjee et al. 2020 |
|  | Stomatal resistance index | CSI3 | ${\text{(T}_{\text{canopy}}\text{ -}\text{T}_{\text{wet}}\text{) }}/{\text{(T}_{\text{dry}}\text{-}\text{T}_{\text{canopy}}})$ | Banerjee et al. 2020 |

**Supplementary Table 2.** List of wheat cultivars with their final disease severity (FDS) based categorization

|  |  | 2017-18 | 2018-19 | Average |  |
| --- | --- | --- | --- | --- | --- |
| S. No | Wheat cultivars | FDS | FDS | FDS | Category* |
|  | VL 829 | 20 | 10 | 15 | **High** |
|  | VL 907 | 15 | 10 | 12.5 | **High** |
|  | HPW 251 | 50 | 40 | 45 | **Low** |
|  | HPW 349 | 30 | 20 | 25 | **Moderate** |
|  | HI 1563 | 20 | 20 | 20 | **High** |
|  | HS 375 | 40 | 40 | 40 | **Moderate** |
|  | HS 507 | 10 | 10 | 10 | **High** |
|  | HD 2932 | 20 | 30 | 25 | **Moderate** |
|  | HD 2967 | 50 | 60 | 55 | **Low** |
|  | HD 3059 | 40 | 40 | 40 | **Moderate** |
|  | HD 3086 | 1 | 5 | 3 | **High** |
|  | HD 3043 | 20 | 20 | 20 | **High** |
|  | PBW 590 | 60 | 50 | 55 | **Low** |
|  | PBW 644 | 20 | 20 | 20 | **High** |
|  | PDW 291 | 10 | 5 | 7.5 | **High** |
|  | PDW 314 | 1 | 1 | 1 | **High** |
|  | Raj 4083 | 20 | 20 | 20 | **High** |
|  | WH 1105 | 30 | 40 | 35 | **Moderate** |
|  | WH 1124 | 1 | 1 | 1 | **High** |
|  | DBW 88 | 40 | 30 | 35 | **Moderate** |
|  | DBW 90 | 1 | 1 | 1 | **High** |
|  | A-9-30-1 | 100 | 95 | 92.5 | **Susceptible** |
|  | HS 240 | 90 | 70 | 80 | **Susceptible** |
|  | PBW 343 | 90 | 80 | 85 | **Susceptible** |

*Category based on average FDS, high (FDS <20%), moderate (FDS : 21–40%), low (FDS : 41–60%) and susceptible (FDS >60%) cultivars.

**Supplementary Table 3.** Hyperparameters and the important variables of 10 models for predicting wheat yield based on sRPI rankings

| S.No. | Model | Stage | Hyperparameters | Important Variables |
| --- | --- | --- | --- | --- |
| 1. | Cubist | 3 | committees = 20 and neighbors = 9 | RWC, P, GBRI, BRD, CI and ExG |
| 2. | RF | 3 | mtry = 51, splitrule = extratrees  and min.node.size = 5 | NDVI, LAI, T,P, and CSI3 |
| 3 | XGB | 2 | nrounds = 51, max_depth = 1, eta = 0.3, gamma = 0, colsample_bytree = 0.6, min_child_weight = 1 and subsample = 1 | LAI, RWC, SI, CWSI and GLAI |
| 4 | RF | 2 | mtry = 29, splitrule = variance and min.node.size = 5 | C, LAI, T, NDVI and G |
| 5 | ELNET | 3 | alpha = 1 and lambda = 0.2846936 | MxEG, NDVI,CWSI, HI and MSI |
| 6 | Cubist | 2 | committees = 1 and neighbors = 5 | CWSI, HI, IG, MSI and RI |
| 7 | XGB | 3 | nrounds = 51, max_depth = 1, eta =  0.3, gamma = 0, colsample_bytree = 0.8, min_child_weight = 1 and subsample = 1 | RI, LAI, MxEG, TCVI and NDVI |
| 8 | SpikeSlab | 3 | vars = 40 | T, LAI,NDVI, P and RI |
| 9 | GPR | 3 | 'sigma’ = 0.01742749 | T, LAI,NDVI, P and RI |
| 10 | Cubist | 1 | committees = 20 and neighbors = 5 | LAI, CWSI, IG, GRRI and RBD |

*P : Photosynthesis, T ; Transpiration, C : Intercellular CO_2_ concentration G : Stomatal conductance*
